# Continuous synthesis of *E. coli* genome sections and Mb-scale human DNA assembly

**DOI:** 10.1038/s41586-023-06268-1

**Published:** 2023-06-28

**Authors:** Jérôme F. Zürcher, Askar A. Kleefeldt, Louise F. H. Funke, Jakob Birnbaum, Julius Fredens, Simona Grazioli, Kim C. Liu, Martin Spinck, Gianluca Petris, Pierre Murat, Fabian B. H. Rehm, Julian E. Sale, Jason W. Chin

**Affiliations:** 1 Medical Research Council Laboratory of Molecular Biology, Cambridge, UK; 2 Wellcome Sanger Institute, Wellcome Trust Genome Campus, Hinxton, UK

## Abstract

Whole-genome synthesis provides a powerful approach for understanding and expanding organism function^1–3^. To build large genomes rapidly, scalably, and in parallel we need: (1) methods for assembling megabases of DNA from shorter pre-cursors; and (2) strategies for rapidly and scalably replacing the genomic DNA of organisms with synthetic DNA. Here we develop bacterial artificial chromosome (BAC) stepwise insertion synthesis (BASIS) - a method for megabase-scale assembly of DNA in *Escherichia coli* episomes. We used BASIS to assemble 1.1 Mb of human DNA containing numerous exons, introns, repetitive sequences, G-quadruplexes, and long and short interspersed nuclear elements (LINEs and SINEs). BASIS provides a powerful platform for building synthetic genomes for diverse organisms. We also develop continuous genome synthesis (CGS) - a method for continuously replacing sequential 100 kb stretches of the *E. coli* genome with synthetic DNA; CGS minimizes crossovers^1,4^ between the synthetic DNA and the genome such that the output for each 100 kb replacement provides, without sequencing, the input for the next 100 kb replacement. Using CGS, we synthesized a 0.5 Mb section of the *E. coli* genome – a key intermediate in its total synthesis^1^ – from five episomes in 10 days. By parallelizing CGS – and combining it with rapid oligonucleotide synthesis and episome assembly^5,6^, and rapid methods for compiling a single genome from strains bearing distinct synthetic genome sections^1,7,8^ – we anticipate it will be possible to synthesize entire *E. coli* genomes, from functional designs, in less than 2 months.

Genome synthesis provides unprecedented opportunities to both define the relationship between the sequences of genomes and the functions they encode, and to generate organisms with new and useful functions. The total synthesis of *Mycoplasma* genomes (0.5-1 Mb) enabled genome minimization^2,3^, and the total synthesis of a recoded *E. coli* genome (4 Mb), created a codon compressed organism that uses 61 codons to make proteins^1^; this organism provided a foundation for creating virus resistant cells, orthogonal genetic systems, and genetically programmed polymer and macrocycle synthesis^9–12^.

Although genome synthesis is still in its infancy, efforts to synthesize other genomes^13^ and strategies to recode^8,13–16^, minimize^13,17^ or rearrange genomes^7,17,18^ are under way. Two central challenges in extending genome synthesis to gigabase-scale genomes, and building genomes rapidly, scalably and in parallel, are: (1) the assembly of megabase-scale sequences of DNA from shorter pre-cursors, and (2) the development of strategies for rapidly, specifically, and efficiently replacing the genomic DNA of organisms with synthetic DNA.

The assembly of large DNA, in episomes, provides a foundational technology for building genomes. Entirely synthetic *Mycoplasma* genomes (0.5-1 Mb) were assembled into yeast episomes, from shorter *in vitro* assemblies, before transfer into *Mycoplasma* cells^2,3^. The ability to synthesize the gigabase-scale genomes of plants and animals, composed of chromosomes that span tens to hundreds of megabases^19^, will require technologies for assembling megabases of DNA that can be used to iteratively replace, or build, the DNA in animal and plant chromosomes in a reasonable number of steps. Notably, the human DNA used for sequencing the essentially complete human genome was primarily captured into BACs in *E. coli*^20^, rather than yeast artificial chromosomes in yeast. Indeed, the repetitive nature of much of the human genome sequence makes it unstable in yeast^21,22^. *E. coli* is an attractive host for assembling large stretches of DNA into episomes, as a starting point for building synthetic Gb-scale genomes. However, current methods for DNA assembly in *E. coli* cannot be iterated^23^, use very small fragments for assembly^24^, use site-specific recombinases that introduce scars^23,25,26^, or require *in vitro* linearization and electroporation of DNA fragments^27,28^. Here we introduce an iterative method that does not require *in vitro* linearization and electroporation of DNA fragments and enables the scarless assembly of approximately 100 kb fragments of human DNA into at least 1.1 Mb of episomally-captured human DNA.

The *E. coli* genome was synthesized by iteratively replacing 100 kb regions of the genome with 100 kb fragments of synthetic, episomal DNA using REXER^1,4^ (Extended Data Fig. 1); five steps of REXER replaced approximately 0.5 Mb of the *E. coli* genome with synthetic DNA in a single strain. Note that there were several steps in the design and synthesis of the *E. coli* genome that preceded the synthesis of genomes with 0.5 Mb synthetic sections from episomes; we discuss these steps and their associated timescales in Supplementary Note 1. The entirely synthetic genome was convergently compiled from seven partially synthetic genomes, and several methods for rapidly and convergently compiling entirely synthetic genomes from synthetic sections of approximately 0.5 Mb have been reported^1,7^. A key challenge in *E. coli* genome synthesis is to reduce the time taken for the synthesis of 0.5 Mb sections of the synthetic genome, starting from episomes bearing 100 kb fragments of synthetic DNA. Here, we introduce CGS, a method to address this challenge.

We first simplify REXER by creating universal spacers that can be used to program the cut site of Cas9 for any step of REXER, regardless of the insert or genomic sequence. Second, we develop conjugation coupled with programmed excision for enhanced recombination (CONEXER), a method that accelerates the integration of synthetic DNA into the genome; using CONEXER we decreased the time for a single integration from 4 days to 1 day. Third, we build on CONEXER to develop BASIS, a method for assembling large synthetic DNA sequences in an episome. Using BASIS, we assembled 1.1 Mb of DNA sequence from a human chromosome in *E. coli*. Fourth, we identify strategies that lead to approximately 80% of the post-CONEXER clones containing the entire desired (>100 kb) region of synthetic DNA. Fifth, we iterate CONEXER, to develop a process for (CGS) that does not require the selection of the correct clones from each step by sequencing. Using CGS, we completely replaced a 0.5 Mb section of the *E. coli* genome with recoded synthetic DNA in just ten days.

## Universal spacers

**Extended Data Fig. 1:**
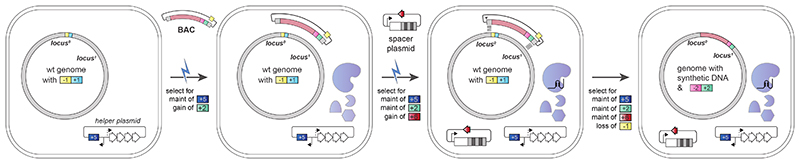
Steps in REXER-mediated integration of ~100 kb of synthetic DNA into the *E. coli* genome using homology region (HR)-specific spacers. REXER allows integration of more than 100 kb of synthetic DNA (pink) into the genome, through replacement of the corresponding genomic DNA. A bacterial artificial chromosome (BAC) containing the synthetic DNA of interest is electroporated into competent cells with a suitably marked genome, the cells also contain a helper plasmid encoding the Cas9 protein and the lambda red recombination components. Selection for the helper plasmid (+5) and the BAC (+2) is applied. A clonal cell is then expanded and induced with arabinose to express the helper plasmid genes and made electrocompetent again. HR-specific spacer arrays (either plasmid-based as shown, or as linear DNA) are then electroporated into the cell; this leads to CRISPR/Cas9 mediated *in vivo* excision of the synthetic DNA, flanked by a double selection cassette (+2/-2) and HRs to the genome, from the BAC. The lambda red recombination machinery then uses the HRs to direct the integration of the excised DNA into the genome. Triangles denote the Cas9 cleavage sites at the HRs (grey boxes) flanking the synthetic DNA. Selection on tetracycline (maintenance of +5), ampicillin (maintenance of +6), chloramphenicol (maintenance of +2), and streptomycin (loss of -1) ensures only cells where the recombination took place over the whole section survive. The selectable markers are +1, blue, *kan^R^* (selected for with kanamycin); -1, yellow, *rpsL* (selected against with streptomycin); +2, green, *cat* (selected for with chloramphenicol); -2, pink, *sacB* (selected against with sucrose); +5, dark blue *tet^R^* (selected for with tetracycline); +6, red *amp^R^* (selected for with ampicillin).

**Fig. 1:**
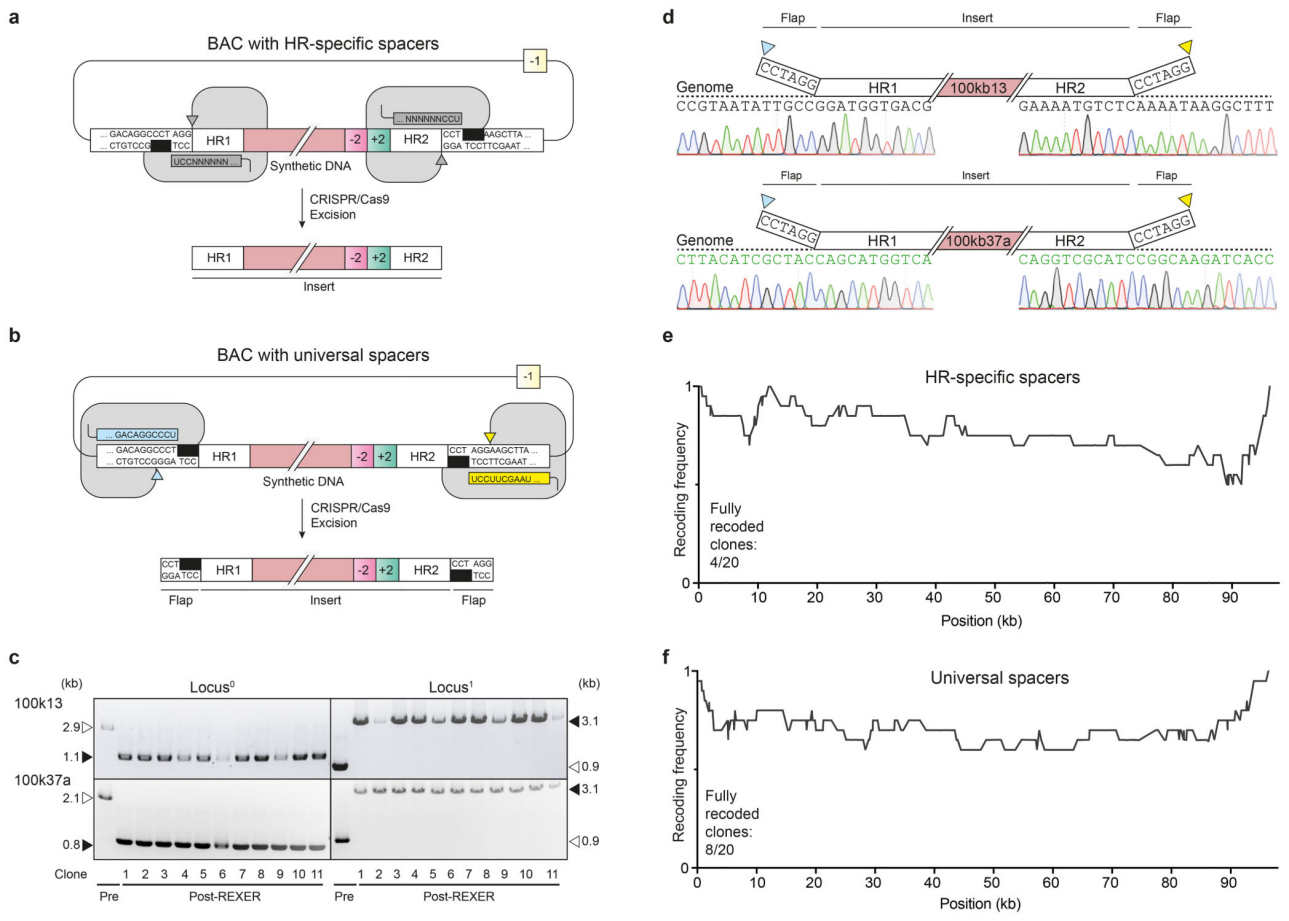
Universal spacers enable scarless replacement of genomic DNA with synthetic DNA. **a**, Spacer RNAs (grey boxes) specific for each homology region (HR), as used in REXER; ‘N’ indicates sequences that are complementary to HR sequences. **b**, Universal spacer RNAs direct Cas9 to the constant sequence of the BAC backbone. In panels **a** and **b**: the selectable markers are -1 (yellow, *rpsL*), +2 (green, *cat*), and -2 (*pink, sacB*). PAM sequences are indicated by a black box. The triangles denote Cas9 cleavage sites. **c**, Verification by PCR of the 5’ and 3’ genomic integration sites after REXER using universal spacers at two genomic loci. A total of 11 post-REXER clones were genotyped for each experiment. The triangles indicate the size of the expected PCR product at each locus before (white) and after (black) REXER. **d**, Sequence verification of the junctions between the insert and the rest of the genome after REXER using universal spacer RNA. The insert and 6 bp non-homology sequences (flap, tilted) are shown above the sequence that is expected for scarless integration (The full dataset is provided in Supplementary Fig. 3). The triangles indicate the Cas9 cut sites. **e, f**, Compiled recoding landscapes of REXER with HR-specific (e) and universal (f) spacers. We performed REXER, replacing 95.6 kb of *E. coli* genomic DNA with synthetic DNA (100k24 from our recoded whole genome synthesis)^1^. A total of 20 post-REXER clones were fully sequenced for each experiment. The compiled recoding landscape graphs show the average frequency at which each recoded codon was integrated across the genomic locus^1,4^. The experiment in **c** was performed in one biological replicate. Gel source data are provided in Supplementary Fig. 1.

REXER (Extended Data Fig. 1) requires a new set of homology region (HR)-specific spacers for each locus that is targeted (Fig. 1a and Supplementary Data 1 and 2); the recent *E. coli* genome synthesis used 78 unique spacers. Each new set of spacers must be designed to avoid undesired genome cleavage, and spacers can be challenging and time-consuming to clone. Moreover, varying the spacer sequence can affect the CRISPR/Cas9 efficiency and thereby lead to potential variations in the efficiency of REXER at distinct genomic loci. Removing the requirement for HR-specific spacer RNA would considerably simplify experimental workflows and accelerate large-scale genome engineering and genome synthesis.

We designed universal spacers (Fig. 1b, Supplementary Fig. 2 and Supplementary Data 3 and 4), which target constant sequences within a BAC backbone, rather than the variable DNA insert, to direct excision of the insert in REXER. Although we minimized the distance of the cleavage site from the junction between the BAC backbone and the insert, cleavage was formally expected to lead to the excision of an insert flanked by 6 bp of the BAC backbone on each end.

We performed REXER – using even and odd BACs bearing synthetic DNA (as used in sequential steps of genome synthesis) – using universal spacers at five loci^1^ (100k13, 100k22, 100k24, 100k28 and 100k37). We confirmed successful integration in 11/11 post-REXER clones at each of these loci by genotyping for loss of the original double-selection cassette from the genome and integration of the new double selection cassette into the genome (Fig. 1c and Supplementary Fig. 2).We sequenced the junctions between the DNA integrated by REXER and the rest of the genome for the 55 post-REXER clones (Fig. 1d and Supplementary Figs. 2 and 3); these experiments demonstrated that the 6 bp mismatched sequences, generated by universal spacer-mediated excision from the BAC, were efficiently and reliably removed in all 55 post-REXER clones. We suggest that the non-homologous ends of the DNA in the BAC may be removed by exonucleases prior to recombination^29^, or by flap endonucleases such as EcoIX during recombination^30^ (through a similar mechanism to that described for FEN1 in eukaryotes^31^).

Additional experiments demonstrated that the compiled post-REXER recoding landscapes^4^, and the fraction of fully recoded clones, are all comparable when using universal spacers or HR-specific spacers (Fig. 1e,f). We conclude that REXER with universal spacers enables the scarless integration of synthetic DNA into the genome with an efficiency comparable to that achieved when using REXER with HR-specific spacers.

## Conexer

**Extended Data Fig. 2:**
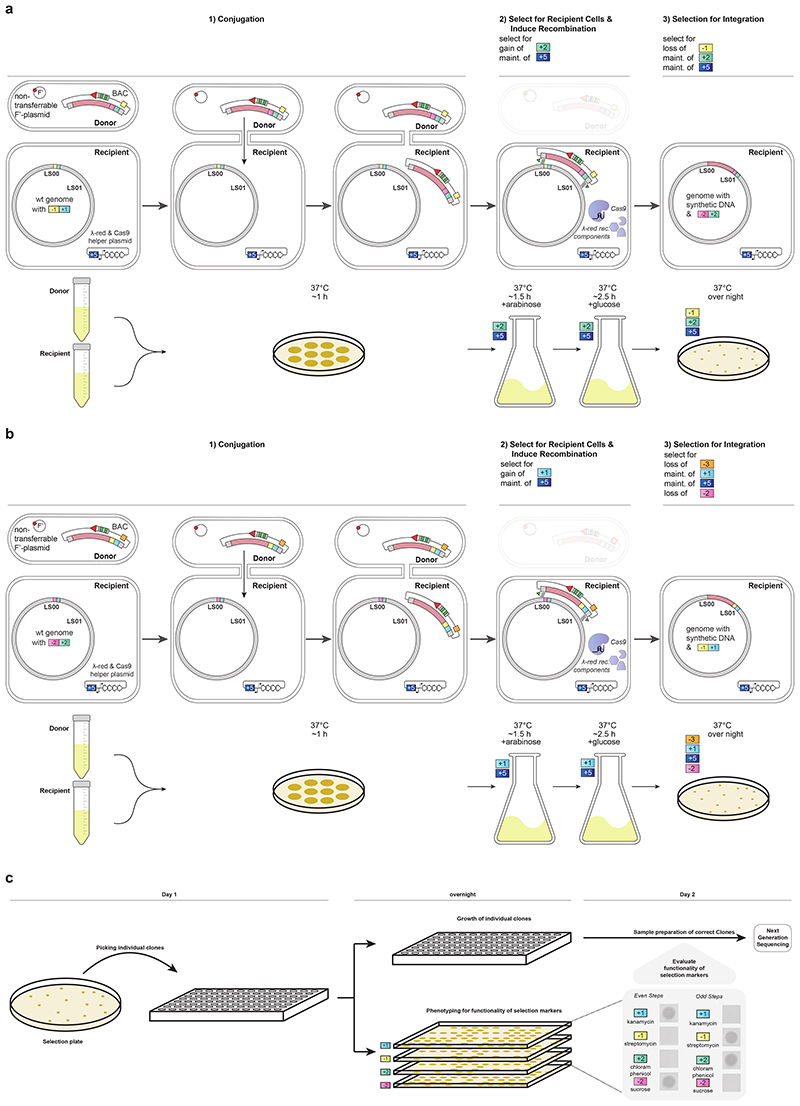
Step-wise depiction of CONEXER procedure. **a**, In an odd step of CONEXER, recipient cells with a +1/-1 double selection cassette in their genome (+1, *kan^R^* (confers growth on kanamycin); -1, *rpsL* (confers sensitivity to streptomycin)) and a tetracycline resistance (+5, *tet^R^* (confers resistance to tetracycline)) conferring plasmid encoding arabinose inducible lambda red components and Cas9 are mixed with donor cells and spotted on an agar plate. Donor cells contain an odd BAC and a non-transferable F’ plasmid. During incubation on the plate (1h, 37 °C) the BAC is conjugated from donor to recipient cells. Subsequently, cells are washed off the plate and inoculated in selective media containing tetracycline (selection for maintenance of +5) and chloramphenicol (selection for gain of +2), this selects for recipient cells that have received the BAC. Arabinose is also added to induce lambda red components and Cas9; excision of the linear DNA and recombination with the genome is induced at this step. Cells are recovered in selective media containing tetracycline (selection for maintenance of +5) and chloramphenicol (selection for maintenance of +2) but no arabinose; this selects for recipient cells that have received the BAC. Finally, cells are plated on selective agar plates containing tetracycline (selection for maintenance of +5) – to select for recipient cells, chloramphenicol (selection for maintenance of +2) – to select for genomic integration of the +2/-2 double selection cassette from the BAC, and streptomycin (selection for loss of -1) – to select for loss of the +1/-1 double selection cassette from the genome and loss of the BAC backbone. **b**, In an even step of CONEXER, recipient cells with a +2/-2 double selection cassette in their genome (+2, *cat* (confers growth on chloramphenicol); -2, *sacB* (confers sensitivity to sucrose) and a tetracycline resistance (+5 *tet^R^* (confers resistance to tetracycline)) conferring plasmid encoding arabinose inducible lambda red components and Cas9 are mixed with donor cells and spotted on an agar plate. Donor cells contain an odd BAC and a non-transferable F’ plasmid. During incubation on the plate (1h, 37 °C) the BAC is conjugated from donor to recipient cells. Subsequently, cells are washed off the plate and inoculated in selective media containing tetracycline (selection for maintenance of +5) and kanamycin (selection for gain of +1); this selects for recipient cells that have received the BAC. Arabinose is also added to induce lambda red components and Cas9; excision of the linear DNA and recombination with the genome is induced at this step. Cells are recovered in selective media containing tetracycline (selection for maintenance of +5) and kanamycin (selection for maintenance of +1) but no arabinose; this selects for recipient cells that have received the BAC. Finally, cells are plated on selective agar plates containing tetracycline (selection for maintenance of +5) – to select for recipient cells, kanamycin (selection for maintenance of +1) – to select for genomic integration of the +1/-1 double selection cassette from the BAC, sucrose (selection for loss of -2) – to select for loss of the +2/-2 double selection cassette from the genome, and 4-CP (selection for loss of -3) – to select for loss of the BAC backbone. **c**, Clones from CONEXER experiments are picked from the selection plate. They are grown up individually in a 96-well plate and phenotyped, for the functionality of selection markers, on agar plates. Subsequently, clones that show the correct growth phenotype (even steps: growth on +1, -2; no growth on -1, +2; odd steps: growth on -1, +2; no growth on +1, -2) are sequenced by NGS. The selectable markers are +1, blue, *kan^R^* (selected for with kanamycin); -1, yellow, *rpsL* (selected against with streptomycin); +2, green, *cat* (selected for with chloramphenicol); -2, pink, *sacB* (selected against with sucrose); -3, orange, pheS* (selected against with 4-chlorophenylalanine); +5, dark blue, *tet^R^* (selected for with tetracycline).

**Fig. 2:**
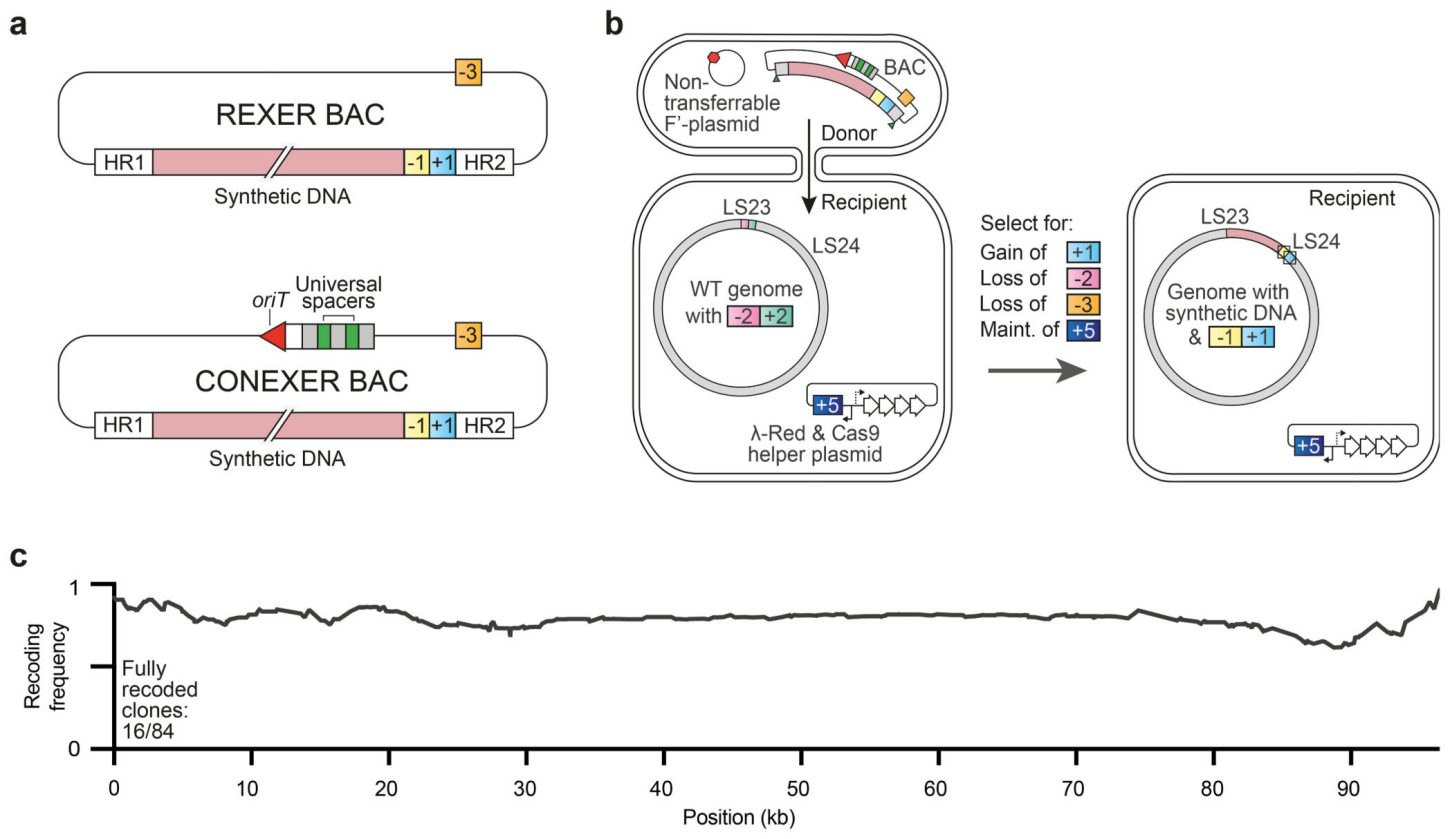
CONEXER is a rapid, simplified, and standardized method for genome synthesis from synthetic DNA in episomes. **a**, REXER and CONEXER BACs. The CONEXER BACs contain a universal spacer array (green bars), origin of transfer (*oriT*, red arrow). **b**, The CONEXER BAC is conjugatively transferred from donor cells to recipient cells with the aid of a non-transferable F’ plasmid (F’). Recipient cells, with an appropriately marked genome (+2/-2 at LS23 is shown), express Cas9 and the λ-red components. Selection for +5 selects recipient cells. Replacement of genomic DNA with synthetic DNA in the recipient cells is then selected for by selecting for gain of +1 and loss of -2; selection for loss of -3 selects for loss of the BAC backbone. In the examples shown in panels **a** and **b**, the selectable markers are +1 (blue, *kan^R^*), -1 (yellow, *rpsL*), +2 (green, *cat*), -2 (*pink, sacB*), -3 (orange, pheS*), and +5 (dark blue, *tet^R^*). Maint., maintenance. WT, wild type. **c**, The compiled recoding landscape of 84 clones from CONEXER with 100k24.

REXER requires two sequential rounds of competent cell preparation and electroporation and it takes 4 days to go from cells with an appropriately marked genome to having clonal colonies for sequencing on a post-REXER agar plate. To accelerate and simplify the introduction of synthetic DNA into the genome, we created BACs in which universal spacer arrays and an *oriT* sequence were integrated into the BAC backbone (Supplementary Data 5, 6, 7). Even BACs contain the +1/-1 double selection cassette adjacent to their synthetic DNA insert (+1, *kan^R^* (confers growth on kanamycin); -1, *rpsL* (confers sensitivity to streptomycin)), and have the -3 selection cassette (-3, pheS* (confers sensitivity to 4-chlorophenylalanine (4-CP))) in their backbone (Fig. 2a and Extended Data Fig. 2b), while odd BACs contain the +2/-2 double selection cassette adjacent to their synthetic DNA insert (+2, *cat* (confers growth on chloramphenicol); -2, *sacB* (confers sensitivity to sucrose), and have the -1 selection cassette in their backbone (Extended Data Fig. 2a).

We mixed donor cells containing an even BAC (with a synthetic DNA insert) and a non-transferable F’ plasmid^1^ with the recipient cells of interest to facilitate conjugative transfer. The recipient cells contain the +2/-2 double selection cassette at the landing site (LS) in their genome and a plasmid encoding arabinose inducible λ-red components and Cas9 protein; this plasmid confers tetracycline resistance (+5, *tet^R^*) to the recipient (Fig. 2b and Extended Data Fig. 2). We selected – on kanamycin and tetracycline – for recipient cells that had received the BAC through conjugative transfer. We turned on the expression of the Cas9 protein and the λ-red recombination components from the helper plasmid in the recipient with arabinose, and the spacers were expressed from the BAC.

We selected, on agar plates containing tetracycline, kanamycin, sucrose, and 4-CP, for recipient cells in which the negative selection markers (-2, sacB, -3, pheS*) had been lost from both the genome and the BAC backbone, and the positive selection marker (+1, *kan^R^*) had been acquired from the BAC (Fig. 2b).

Using this one-day universal protocol (Extended Data Fig. 2) we introduced synthetic DNA (a completely recoded fragment 24; 96 kb) in place of the corresponding genomic DNA in the recipient cell. We picked colonies and tested for +1, -1, +2, and -2 by examining their growth phenotype on kanamycin, streptomycin, chloramphenicol and sucrose (Extended Data Fig. 2c). Only clones with the correct phenotype for each of the four markers were sequenced. In 19% of the resulting clones the synthetic DNA had completely replaced the corresponding genomic sequence (Fig. 2c); the remaining clones were chimeras between synthetic and natural sequence, resulting from crossovers, as previously observed for REXER^1,4^. We used an analogous process for CONEXER with odd BACs (Extended Data Fig. 2a). We confirmed using sequencing, that colonies with the complete set of correct phenotypes had undergone marker swap (Supplementary Fig. 4). We named our accelerated approach CONEXER.

## Assembly of megabase-scale human DNA in episomes

**Extended Data Fig. 3:**
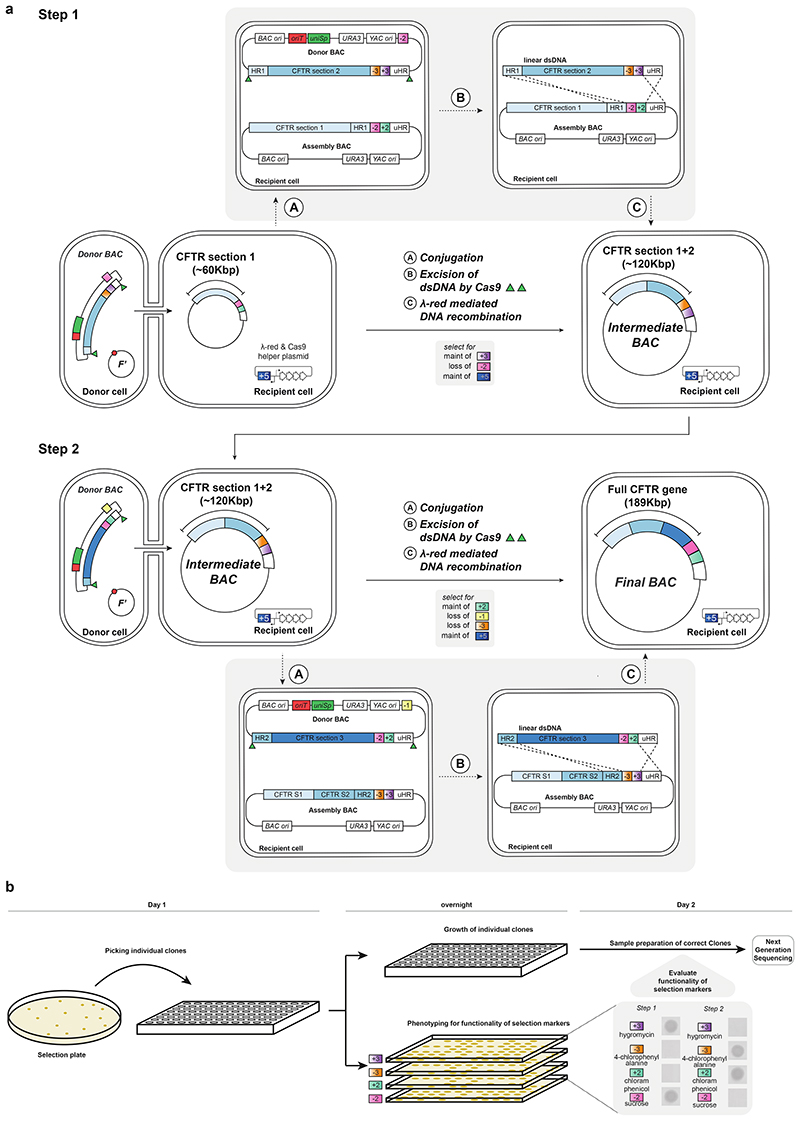
*CFTR* assembly via BASIS. **a**, Recipient cells containing a BASIS assembly BAC with the first section of the *CFTR* gene and a plasmid encoding Cas9 and lambda red components were mixed with donor cells. Donor cells contained a BASIS BAC encoding the second section of the *CFTR* gene and a non-transferable F’ plasmid. The donor BAC was conjugated to the recipient cell (A) and recipient cells selected for on tetracycline (+5, *tet^R^* (confers resistance to tetracycline)). Upon induction of protein expression from the helper plasmid, linear dsDNA was excised from the donor BAC (B). The excised DNA inserts into the assembly BAC between HR1 and uHR. Selection on hygromycin (selection for gain of +3) – to select for gain of the selection cassette from the donor BAC, sucrose (selection for loss of -2) – to select for loss of the selection cassette from the assembly BAC and loss of donor BAC backbone, tetracycline (selection for maintenance of +5) – to select for maintenance of the helper plasmid, ensured that only cells with the correctly assembled BAC survive. In step 2, recipient cells containing a BAC with the first and second section of the *CFTR* gene and a plasmid encoding Cas9, and lambda red components were mixed with donor cells. Donor cells contained a BASIS BAC encoding the third section of the *CFTR* gene and the non-transferable F’ plasmid. The donor BAC was conjugated to the recipient cell (A) and recipient cells selected for on tetracycline (+5, *tet^R^* (confers resistance to tetracycline)). Upon induction of protein expression from the helper plasmid, linear dsDNA was excised from the donor BAC (B). The excised DNA inserts into the assembly BAC between HR2 and uHR. Selection on chloramphenicol (selection for gain of +2) – to select for gain of the selection cassette from the donor BAC), 4-CP (loss of -3) – to select for loss of the selection cassette from the assembly BAC, streptomycin (loss of -1) – to select for loss of the donor BAC backbone, and tetracycline (maintenance of +5) – to select for maintenance of the helper plasmid, ensured that only cells with the correctly assembled BAC survive. **b**, Clones from BASIS experiments were picked from the selection plate. They were grown up individually in a 96-well plate and phenotyped for the functionality of selection markers on agar plates. Subsequently, clones that showed the correct growth phenotype and in some cases genotype for the assembly junctions by PCR (step 1: growth on hygromycin, growth on sucrose; no growth on 4-CP, no growth on chloramphenicol, and genotyping for insertion of the second section of *CFTR* (for primers see Supplementary Data 2); step 2: growth on 4-CP, growth on chlopramphenicol; no growth on hygromycin, no growth on sucrose) were sequenced by NGS. The selectable markers are +3, purple, *hyg^R^* (selected for with hygromycin); -3, orange pheS* (selected against with 4-chlorophenylalanine); +2, green, *cat* (selected for with chloramphenicol); -2, pink, *sacB* (selected against with sucrose); -1, yellow *rpsL* (selected against with streptomycin); +5, dark blue, *tet^R^* (selected for with tetracycline).

**Fig. 3:**
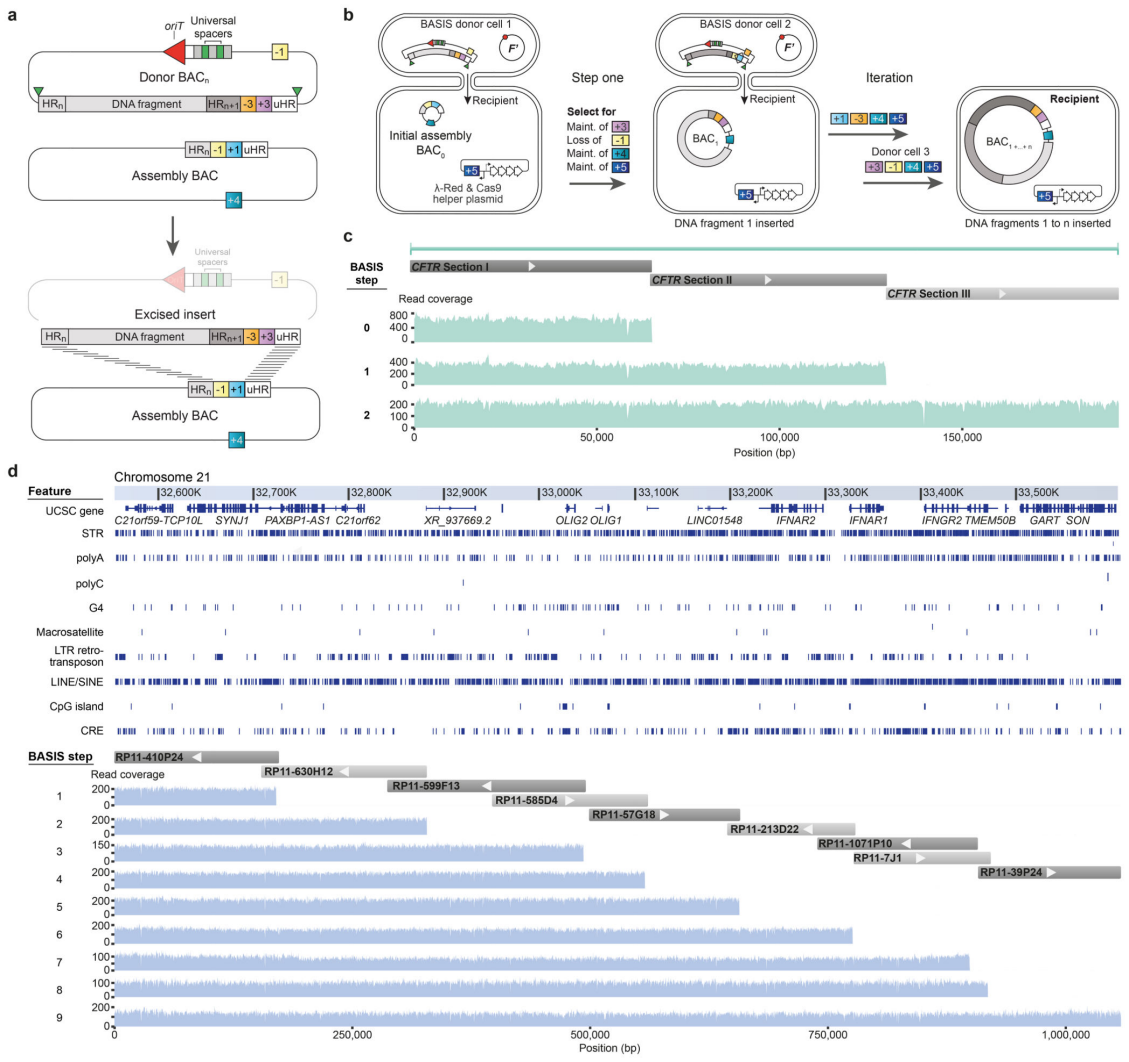
Megabase-scale assembly of DNA using BASIS. **a**, Donor and assembly BACs for BASIS. uHR is the universal homology region. HRn is an HR specific for the nth step of insertion. The donor BAC insert contains HRn+1, which serves as HRn for the (n+1)th step. The green triangles indicate the cut sites for Cas9 excision. **b**, The BASIS workflow. The donor BAC is delivered by conjugative transfer to the recipient cell. The insert is excised from the donor BAC and inserted into the assembly BAC, as shown in (**a**). Iteration, using alternating sets of markers, directs the insertion of n DNA fragments into the assembly BAC. In panels **a** and **b** the selectable markers are +1 (blue, *kan^R^*), -1 (yellow, *rpsL*), +3 (purple, *hygro^R^*), -3 (orange, pheS*), +4 (petrol, *gent^R^*), and +5 (dark blue, *tet*^R^). **c**, Assembly of the full-length *CFTR* gene through BASIS using three BACs and verification using next generation sequencing (NGS). The sequencing coverage tracks for each step of BASIS are shown in a vertical stack; read coverage (y-axis) is plotted against the position in base pairs (bp) from the start of the first insert (x-axis). **d**, BASIS assembly of the 1.1 Mb target region of chromosome 21. The top blue bar indicates the region targeted for assembly. UCSC database genes are indicated. The vertical blue lines indicate the positions of other features, including: short tandem repeats, guanosine quadruplexes (G4), long terminal repeats (LTR) retrotransposons, LINEs, SINEs, and cis regulatory elements (CRE). The RP number and the grey bars indicate the BAC from which the insert was derived. The sequencing coverage track for each assembly step is shown in a vertical stack, as in panel **c**.

We hypothesized that the principles that we had established for CONEXER might be extended to realize the scarless assembly, through iterative insertion, of megabases of DNA into episomes in *E. coli*. We designed an assembly BAC in which to iteratively insert and assemble DNA (Fig. 3a). This BAC contains approximately 60 bp of sequence homologous to one end of the next sequence to be inserted (HR1); this is immediately followed by a positive and negative selection cassette and a universal HR (uHR), which is complementary to the other end of the sequence to be inserted. We also designed donor BACs containing universal spacers and *oriT* (Fig. 3a). In the donor BACs, HR1 is within the 5’ end of the next DNA sequence to be inserted into the assembly BAC; this DNA sequence is followed at its 3’ end by a distinct positive and negative selection cassette and a uHR. Each step of the assembly (Fig. 3a,b) proceeds by conjugative transfer of the donor BAC into recipient cells containing the assembly BAC, Cas9-mediated excision of the sequence from HR1 to the universal homology region from the donor BAC, and λ-red-mediated insertion of this sequence into the assembly BAC (Extended Data Fig. 3). Selection for the loss of the negative selection markers on the assembly BAC and gain of the positive marker from the sequence excised from the donor BAC, selects for cells containing the assembly BAC with the correct insertion. Cells containing the new assembly BAC provide the input for the next step of insertion. We named our approach BASIS.

**Extended Data Fig. 4:**
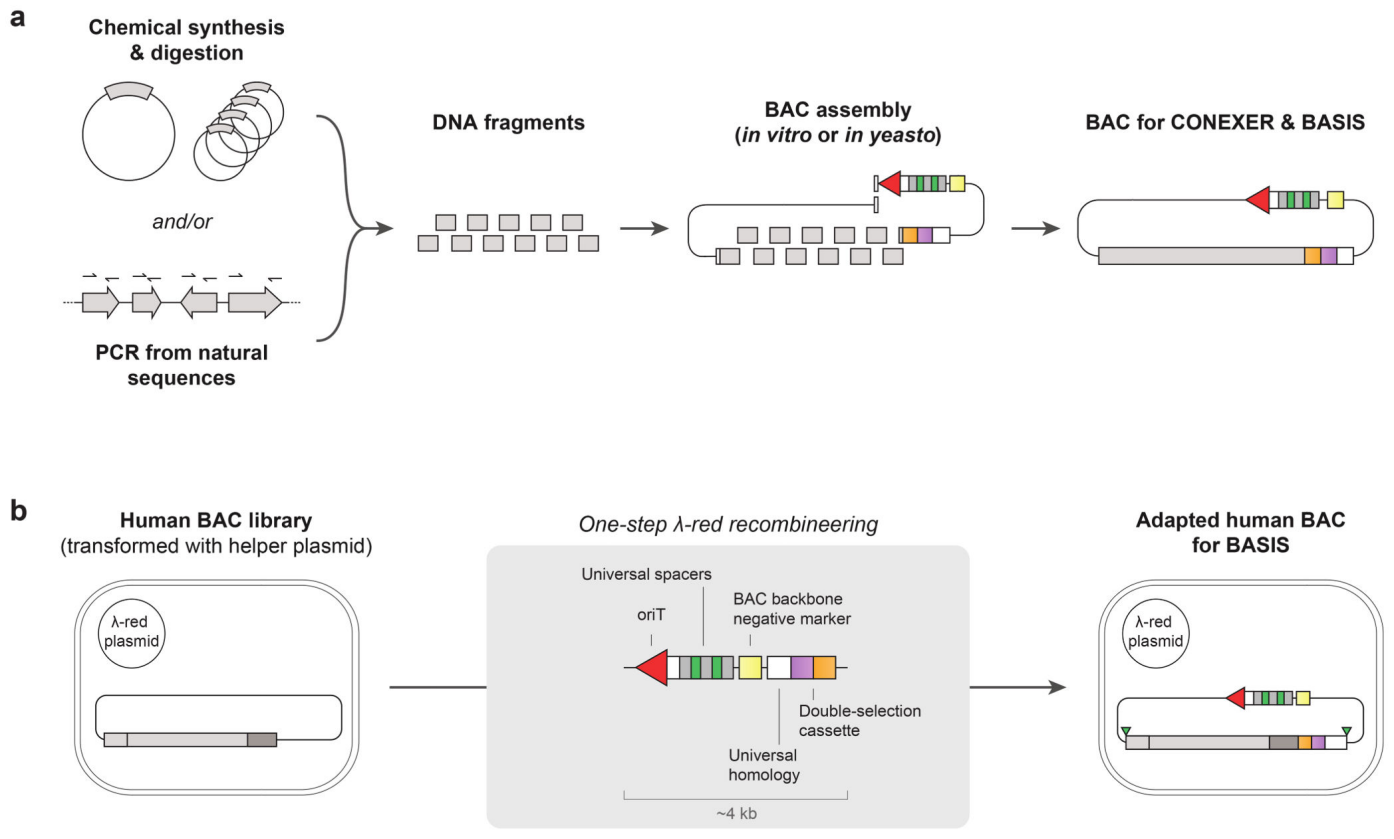
Assembly of BACs to be used for CONEXER and BASIS, and adaptation of existing BACs derived from human BAC libraries. **a**, BAC assembly from DNA fragments of 3-10 kb; these fragments may be derived from chemically synthesized oligonucleotides and/or may be amplified from natural sequences. 10 kb DNA fragments are assembled with established methods, either *in vitro* or *in vivo* by yeast assembly^1–3^. All fragments are assembled with the BAC backbone, which contains components required for subsequent CONEXER or BASIS steps (universal spacers, origin of transfer, marker cassettes). **b**, BACs used for assembly of a megabase-scale human genomic section are derived from human BAC libraries. The sequence of human DNA in these BACs overlap with each other, and these overlaps constitute the homology regions exploited for assembly. The universal spacer cassette, the origin of transfer, the universal homology region, and appropriate selection markers were introduced into BACs from the human BAC library, by one-step λ-red recombineering, to generate BASIS BACs.

We demonstrated the assembly of a 208 kb BAC containing the 189 kb human cystic fibrosis transmembrane regulator (*CFTR*) gene with its endogenous promoter^32^ by two steps of BASIS (Extended Data Fig. 3 and Supplementary Note 2), using three BACs that each contained approximately 60 kb fragments of the gene (Supplementary Data 8-10).We assembled each of these BACs in yeast from 10-12 PCR products, amplified from the human genome, and BAC backbone fragments. This is one of several well-established strategies for assembling BACs, from synthetic DNA or natural DNA, at this scale (Extended Data Fig. 4). We characterized clones of the final BASIS assembly with the correct set of phenotypes (Methods and Extended Data Fig. 3b) by short and long-read next generation sequencing (NGS) and confirmed an error-free BASIS assembly with respect to the input BACs (Fig 3c and Supplementary Data 11 and 12).

**Extended Data Fig. 5:**
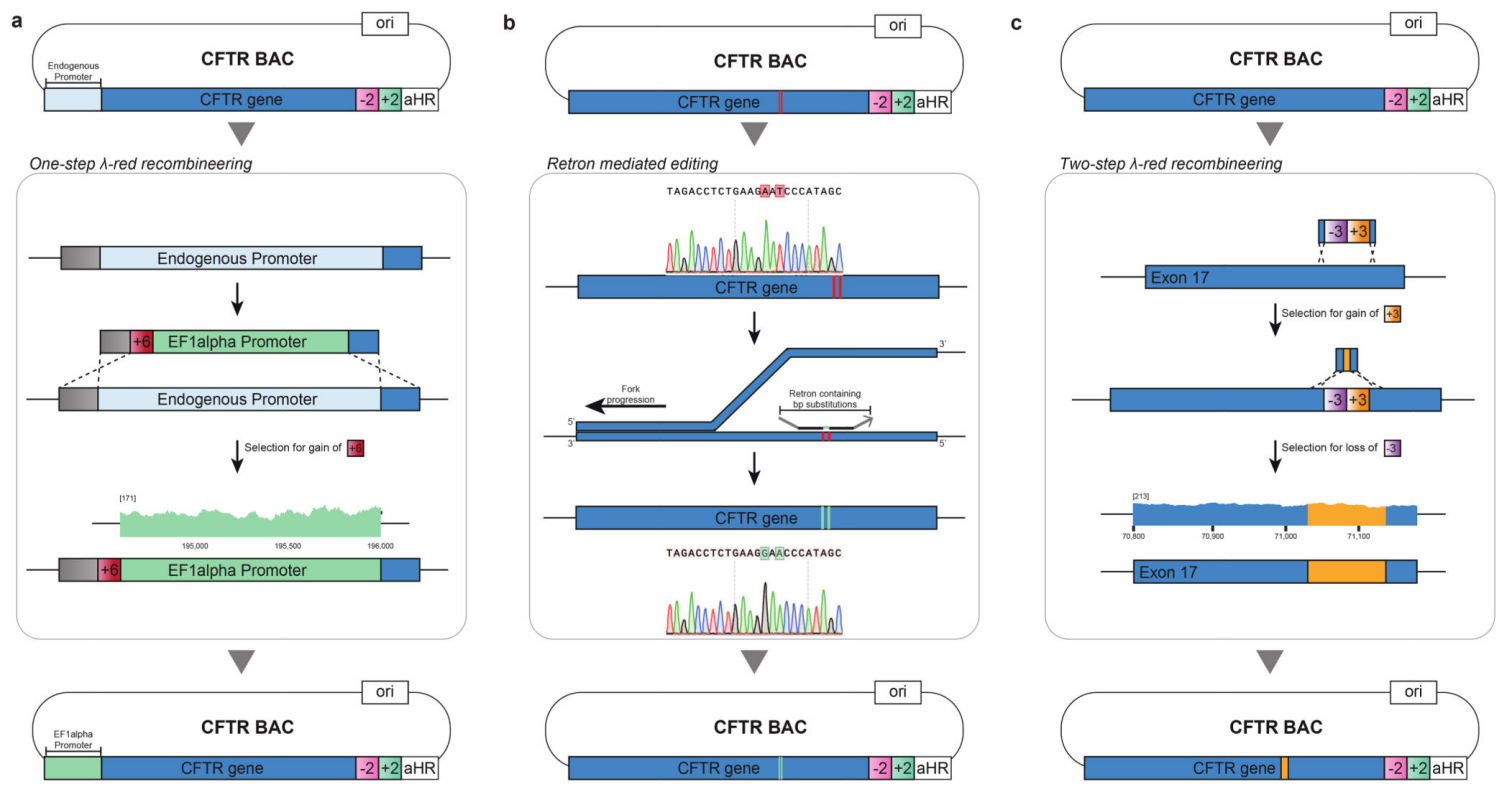
BACs produced by BASIS can be extensively modified by lambda red recombineering and retron-mediated editing – to generate insertions, replacements, and edits. **a**, For expression in human tissue culture, the endogenous *CFTR* promoter was replaced with an EF1alpha constitutive promoter using λ-red recombineering. To this end the EF1alpha promoter was coupled to an ampicillin resistance gene (+6, red *amp^R^* (confers resistance to ampicillin)). Following recombineering cells were selected on ampicillin (selection for gain of +6) – to select for replacement of the promoter. Sequence coverage of the EF1alpha prompter is shown (maximum coverage indicated in brackets). **b**, BACs produced by BASIS can be precisely edited using retron-mediated editing. A single strand binding protein and a retron containing the desired base pair substitutions were expressed in target cells containing the BAC. During replication annealing of the retron to the lagging strand led to the desired edits. We corrected two point mutations in exon 15 of the *CFTR* gene (Methods). Sanger sequencing traces of the region containing the point mutations are shown before (top - red) and after (bottom - green) editing. **c**, To distinguish BAC encoded *CFTR* from the endogenous gene an HA-tag was inserted into exon 17 of the *CFTR* gene on the BAC. This tag is known to be tolerated in the cDNA of *CFTR*^4^. First, a double selection cassette (+3, orange *hyg^R^* (confers resistance to hygromycin); -3, purple pheS* (confers sensitivity to 4-CP)) was inserted into the locus of interest. Following recombineering cells were selected on hygromycin (selection for gain of +3) – to select for insertion of the double selection cassette. Subsequently, λ-red recombineering was used to replace the double selection cassette with an HA-tag. Following recombineering cells were selected on 4-CP (selection for loss of -3) – to select for replacement of the double selection cassette. Sequence coverage of exon 17 containing the HA-tag is shown (maximum coverage indicated in brackets).

To demonstrate the facile modification of BACs assembled by BASIS in *E. coli*, we modified a *CFTR* BAC (Extended Data Fig. 5). We used λ-red recombination to introduce an EF-1α promoter in place of the endogenous promoter and inserted an HA-tag sequence at the end of exon 17. We also used retron editing to correct two point mutations already present in the input BACs (Supplementary Data 13 and 14); these mutations were probably introduced during PCR amplification of genomic DNA. These experiments demonstrated that we can rapidly insert synthetic sequences, and replace and edit sequences, in large episomes assembled by BASIS.

**Extended Data Fig. 6:**
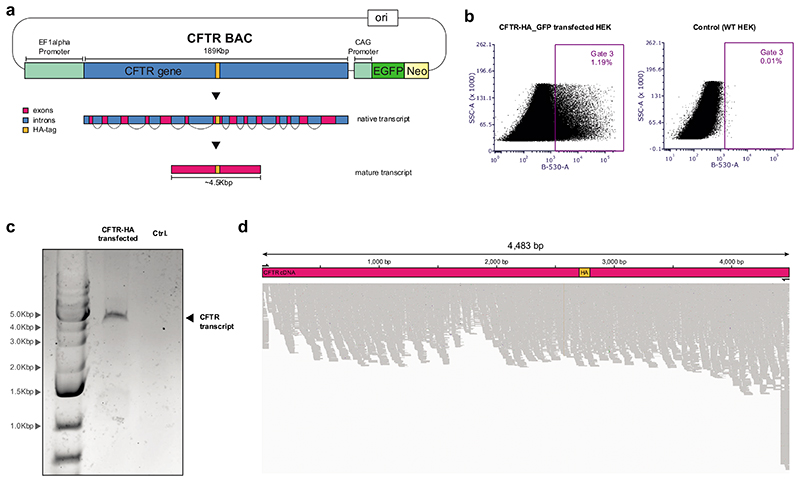
Expression of *CFTR* from a modified BASIS BAC in mammalian cells. **a**, Schematic of a 208 kb *CFTR* BAC: the entire human *CFTR* gene with an HA-tag (yellow-bar) was expressed under a constitutive EF1alpha promoter. The native transcript, containing exons and introns is spliced to form the mature transcript of approximately 4.5 kb in length. Additionally, *eGFP* and a Neomycin (Neo) resistance are expressed from a constitutive CAG promoter. **b**, FACS plots from human embryonic kidney (HEK293) cells transfected with the *CFTR* BAC (*CFTR*-HA_GFP) and a control (WT). On the x-axis the fluorescence intensity from GFP protein is displayed. On the y-axis side-scattering in displayed. Approximately, 1.19% of cells transfected with the *CFTR* BAC are positive for GFP. **c**, PCR was performed on cDNA from cells with and without the transfected *CFTR* BAC. The band (around 4.5 kb) corresponding to an amplicon of *CFTR* transcript is specific to cells transfected with the *CFTR* BAC. Band sizes of the ladder are indicated to the left (grey arrows). The experiment in **c** was performed in one biological replicate. **d**, NGS of the PCR product shown in (**c**) demonstrates that *CFTR* has been transcribed and processed properly in cells after transfection. The presence of an HA-tag in the transcript shows that the transcript is derived from the BASIS BAC encoded *CFTR*. Increased coverage at the flanks likely stems from sequencing of the primers.

We transfected a modified *CFTR*-containing BAC into human embryonic kidney (HEK293) cells and collected cells that were green as a result of a GFP gene in the BAC (Extended Data Fig. 6, and Supplementary Fig. 5); the 4.5 kb *CFTR* cDNA could be amplified from these cells but could not be amplified from untransfected HEK293 cells. Sequencing the amplicon confirmed that the *CFTR* gene from the BAC had been correctly spliced to the mature *CFTR* transcript. These experiments demonstrated that BASIS BACs containing large DNAs provide a facile platform for rapid modification in *E. coli* and expression in human cells.

**Extended Data Fig. 7:**
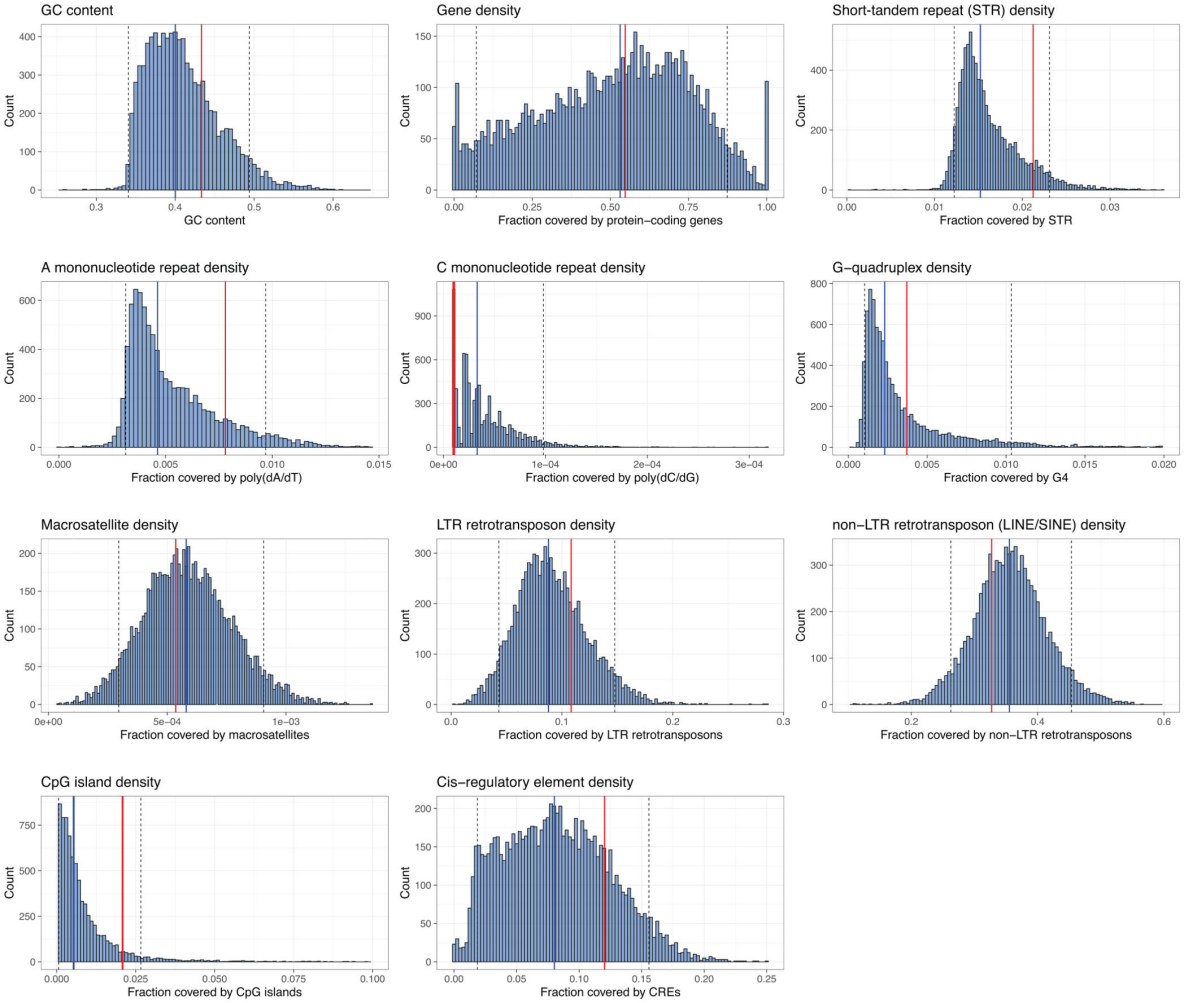
Analysis of genomic features in the 1.1 Mb target region on chromosome 21. Analysis of distribution of genomic features in the 1.1 Mb target region of the human genome assembled by BASIS. The genomic features in this region are compared to the distribution of the genomic feature throughout the whole genome, computed in 1 Mb windows. The red line indicates the fraction of each genomic feature for the 1.1 Mb target region. The blue line indicates the median for each genomic feature for the whole genome. The dotted lines represent the 5^th^ and 95^th^ percentile of the distribution.

We next demonstrated that BASIS can be used to assemble large sections of human genomic DNA, including exonic, intronic and intergenic regions, into a single episome. We focussed on assembling a 1.1 Mb region of the human genome within chromosome 21. This region includes repetitive sequences, G-quadruplexes, and LINEs/SINEs (Fig. 3d). Further analysis indicated that these features, as well as gene density, occur within this 1.1 Mb region at comparable levels to their median levels in the genome (Extended Data Fig. 7).

We started with a library of human BACs used for the essentially complete sequencing of the human genome^33^, each of these human BACs contains approximately 170 kb of human DNA. We used one step of λ-red recombination to convert nine members of the human BAC library, covering the targeted 1.1 Mb region of chromosome 21, into donor BACs for BASIS; this step introduced a positive- and negative-selection cassette, uHR, *oriT*, and universal spacers (Extended Data Fig. 4 and Supplementary Data 15-17). The human DNA sequences in these BACs overlap by 14-122 kb.

We performed nine steps of BASIS to assemble an episome containing 1.1 Mb of human DNA in *E. coli*. We identified clones with the correct marker swap, on the basis of their phenotypes and/or genotypes, after each step of BASIS (Methods). We verified the correct assembly of these clones using short-read sequencing (Fig. 3d) and used a correctly assembled clone as the input for the next step of BASIS. The final assembly was characterized by short- and long-read sequencing (Fig. 3d). Across the 1.1 Mb sequence, we observed four single-base insertions or deletions in mononucleotide repeats, and one 69 base contraction in a TA-rich repeat (Supplementary Data 12, 18 and 19). These repeat sequences are of variable length in the human population^34^ as a result of polymerase slippage during replication, which increases the mutation rate by orders of magnitude at these loci to 10^-5^ to10^-3^ per locus per generation^35^; as these sequences go through approximately 300 replication cycles in their generation by BASIS, it is not surprising that some of the diversity found in humans is regenerated in the assembly. We conclude that BASIS enables the high-fidelity, megabase-scale assembly of human DNA with representative gene density, repetitive sequences, G-quadruplexes, and LINEs/SINEs.

## Crossover minimization

In our total synthesis of the *E. coli* genome each step of REXER was followed by genome sequencing to identify clones in which genomic DNA had been replaced with synthetic DNA across the entire 100 kb region targeted. One such clone was then used as the input for the next round of REXER^1,4^. This was necessary because crossovers occurred between genomic DNA and the synthetic DNA, such that only approximately 20% of the clones from each step had replaced all 100 kb of genomic DNA with synthetic DNA. Thus, without identifying a correct clone by sequencing at each step, five steps would yield fully recoded clones with a frequency of no more than 3x10^-4^, and therefore tens of thousands of clones would need to be sequenced to identify a single clone with the correct sequence. While sequencing after each step of REXER was necessary to complete the synthesis it slowed progress and increased cost.

We envisioned iterating CONEXER by directly using an unsequenced pool of clones from one CONEXER as the direct input for the next CONEXER. To do this, we set out to identify factors that substantially decrease the crossovers between genomic DNA and synthetic DNA, and thereby increase the fraction of clones in which the genomic DNA had been completely replaced with synthetic DNA in a single step of CONEXER.

**Fig. 4:**
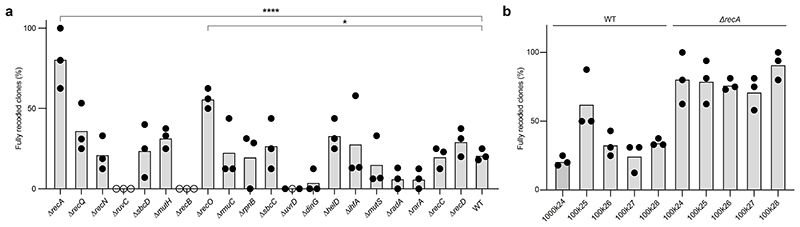
Deletion of *recA* from the host genome increases the fraction of fully recoded clones in CONEXER mediated genomic replacement. **a**, Screening of gene deletion strains in CONEXER mediated replacement of genomic fragment 100k24 for increased frequency of fully recoded clones. We performed a one-way analysis of variance comparing each condition with the WT and corrected for multiple hypotheses using the conservative Bonferroni-correction. Deletions of *recA (P* < 0.0001) and *recO (P* = 0.04) significantly increased the fraction of clones with fully synthetic sequences. Data are represented as the mean of n = 3 independent biological replicates. **b**, Deletion of *recA* increases the fraction of fully recoded clones across several 100 kb fragments (100k24 - 100k28) after CONEXER.

We identified 20 host-factors^36^ that are involved in DNA repair, replication, and recombination to test for their contribution to CONEXER (Fig. 4a). We deleted each of these factors in *E. coli* (Supplementary Data 20) and performed CONEXER with 100k24 in the resulting deletion strains. These experiments identified that deletions of *recA (∆recA*; P < 0.0001) and *recO* (P = 0.04) significantly increased the fraction of clones with a fully synthetic sequence in their genome (Fig. 4a and Supplementary Fig. 6). *∆recA* increased the percentage of clones with fully synthetic DNA from 20% to approximately 80% for 100k24 (Fig 4a). We observed similar substantial increases in the percentage of clones with fully synthetic DNA across several other 100 kb regions, underscoring the generality of our observations (Fig 4b).

**Extended Data Fig. 8:**
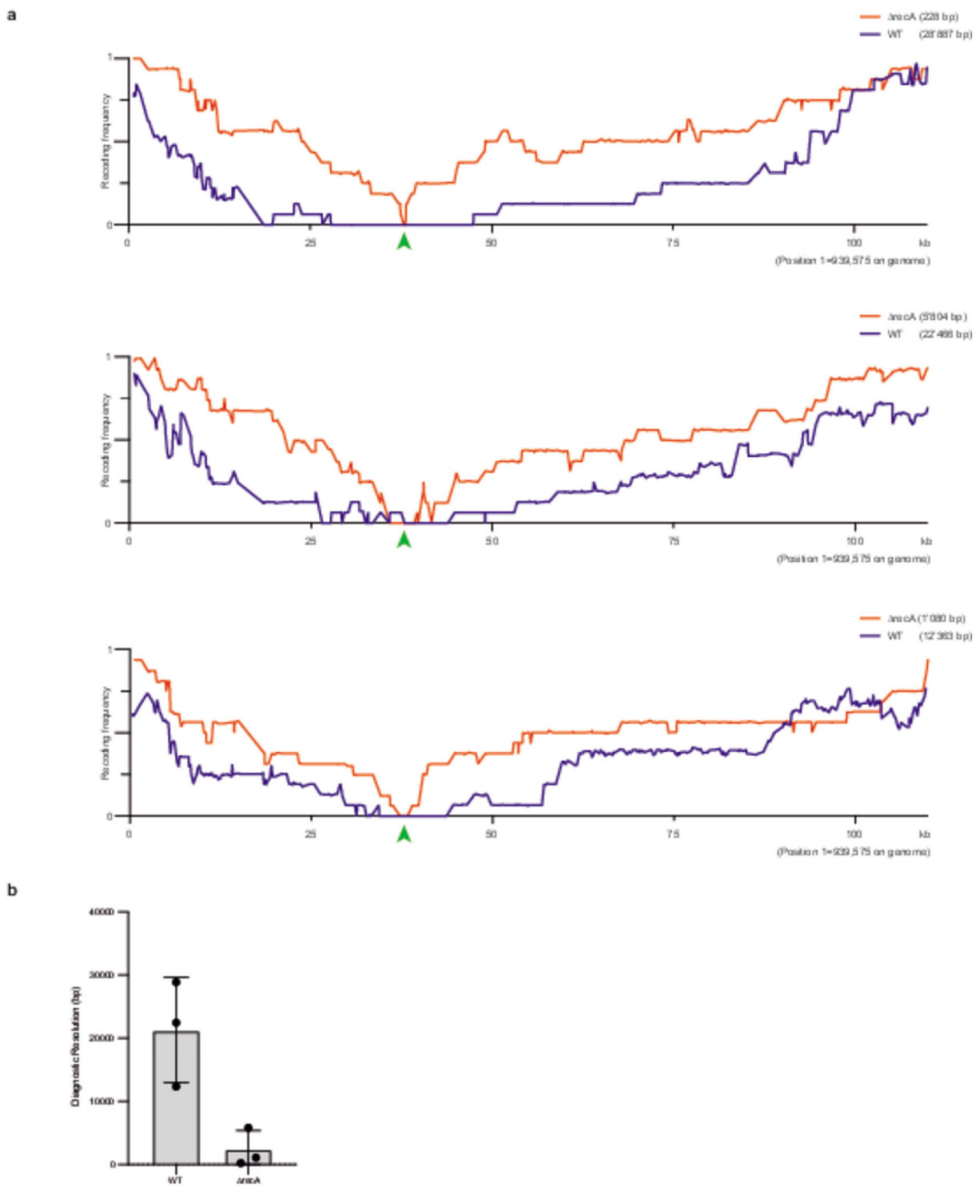
CONEXER experiments on sequences that cannot be replaced by recoded synthetic DNA. **a**, Codons in yceQ at positions 37’213, 37’227, 37’251 (indicated by green arrow), within 100k09, cannot be replaced by recoded synthetic DNA in which TCG codons are replaced with AGC, TCA codons are replaced with AGT, and TAG codons are replaced with TAA. Each graph shows a compiled recoding landscape in WT genetic background (blue) and ∆*recA* (red). Landscapes were generated by sequencing 13-16 clones from an independent CONEXER experiment with a CONEXER BAC bearing recoded sequence for 100k09. The resolution of the diagnostic landscapes (distance between the positions of the first and last event with recoding frequency 0) is indicated in parentheses. Three independent replicates of each experiment are shown. **b**, Bar graph of the diagnostic resolution of CONEXER experiments in section 100k09 in WT and ∆*recA* conditions; data are from n=3 independent biological replicates shown in panel **a**. Data are represented as mean +/- standard deviation. The ∆*recA* condition is significantly better than the WT at localizing disallowed synthetic sequences (two-sided unpaired t test: p-value = 0.021). We note that previous experiments by REXER in WT background yielded a resolution of >20’000 bp^1^.

We have previously shown that synthetic sequences that are not tolerated by the cell can be localized by visualizing the crossovers that occur in post-REXER compiled recoding landscapes^1,4^. Here we showed that CONEXER in ∆*recA* cells enables the localization of a known problematic synthetic sequence in 100k09 with a greater precision than REXER or CONEXER in *recA* positive cells (Extended Data Fig. 8). We conclude that our procedure is probably at least as good as REXER in localizing problematic synthetic sequences within 100 kb regions of synthetic DNA.

## Rapid and continuous genome synthesis

**Extended Data Fig. 9:**
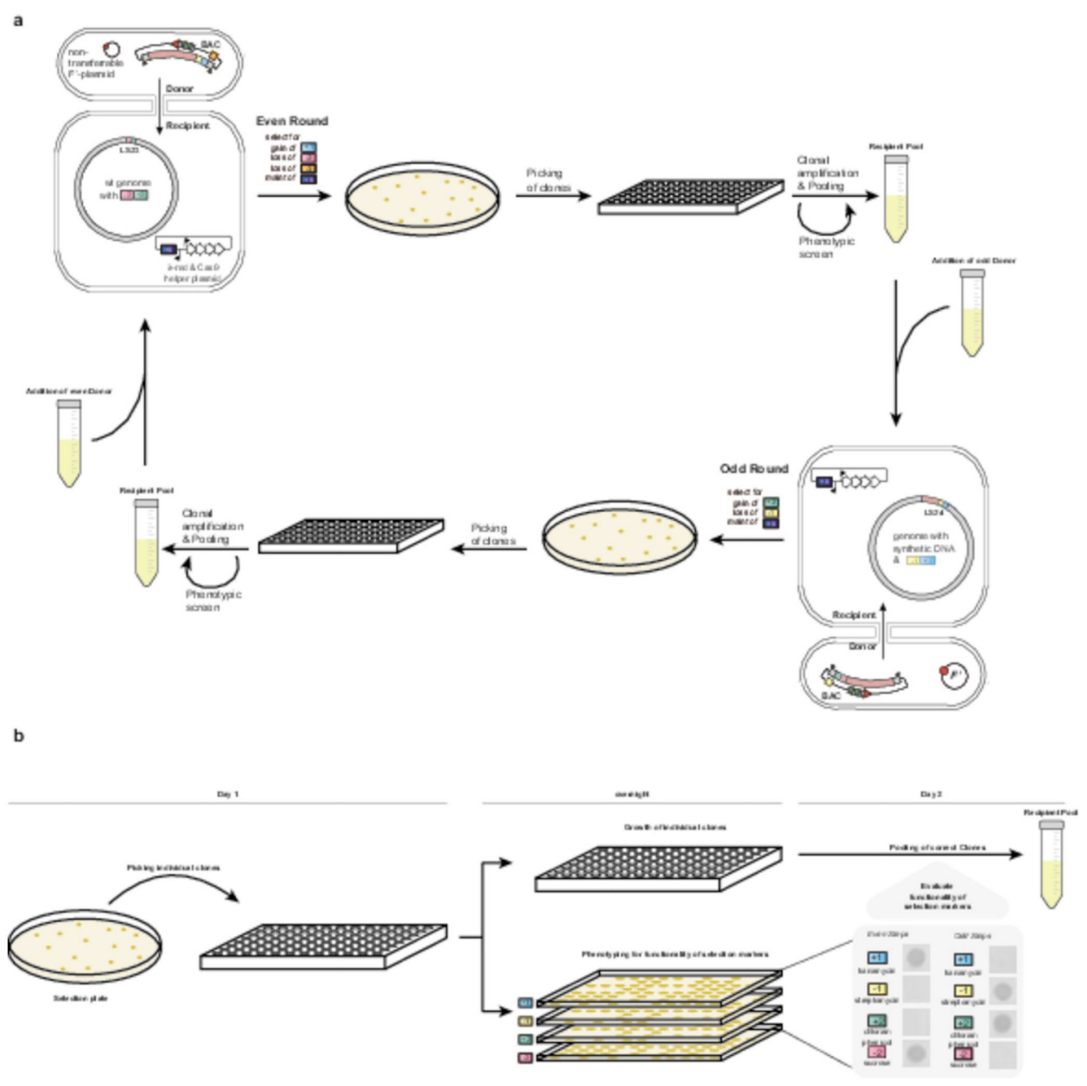
Continuous genome synthesis method. Alternating even and odd rounds of CONEXER for continuous replacement of genomic DNA with synthetic DNA. **a**, Following an even round of CONEXER, clones are picked from an appropriate selection plate containing kanamycin (selection for gain of +1), sucrose (selection for loss of -2), 4-chlorophenylalanine (selection for loss of -3), and tetracycline (selection for maintenance of +5). Clones are amplified overnight and in parallel undergo a phenotypic screen. Amplified clones with all the correct phenotypes are pooled. This pool serves directly as the recipient for an odd round of CONEXER. After an odd round of CONEXER clones are picked from an appropriate selection plate containing chloramphenicol (selection for gain of +2), streptomycin (selection for loss of -1), and tetracycline (selection for maintenance of +5). Clones are amplified overnight and in parallel undergo a phenotypic screen. Amplified clones with all the correct phenotypes are pooled. This pool serves directly as the recipient for an even round of CONEXER. Cycling through even and odd rounds of CONEXER lead to continuous synthesis of a synthetic genome from the corresponding BACs. **b**, Colonies obtained from a step of CONEXER are picked from the selection plate. They are grown up individually in a 96-well plate and phenotyped for the functionality of selection markers on agar plates. Subsequently, clones that show the correct growth phenotype (even steps: growth on kanamycin (selection for +1), growth on sucrose (selection against -2); no growth on streptomycin (selection against -1), no growth on chloramphenicol (selection for +2); odd steps: growth on streptomycin (selection against -1), growth on chloramphenicol (selection for +2); no growth on kanamycin (selection for +1), no growth on sucrose (selection against -2)) are pooled into one culture. This culture immediately serves as the recipient strain for the next step of CONEXER. The selectable markers are +1, blue, *kan^R^* (selected for with kanamycin); -1, yellow, *rpsL* (selected against with streptomycin); +2, green, *cat* (selected for with chloramphenicol); -2, pink, *sacB* (selected against with sucrose); -3, orange, *pheS* (selected against with 4-chlorophenylalanine); +5, dark blue, *tet^R^* (selected for with tetracycline).

Next, we examined whether we could directly use the output from one round of CONEXER – without identifying an individual, fully recoded, clone by sequencing – as the input for the next round of CONEXER (Extended Data Fig. 9a).

**Extended Data Fig. 10:**
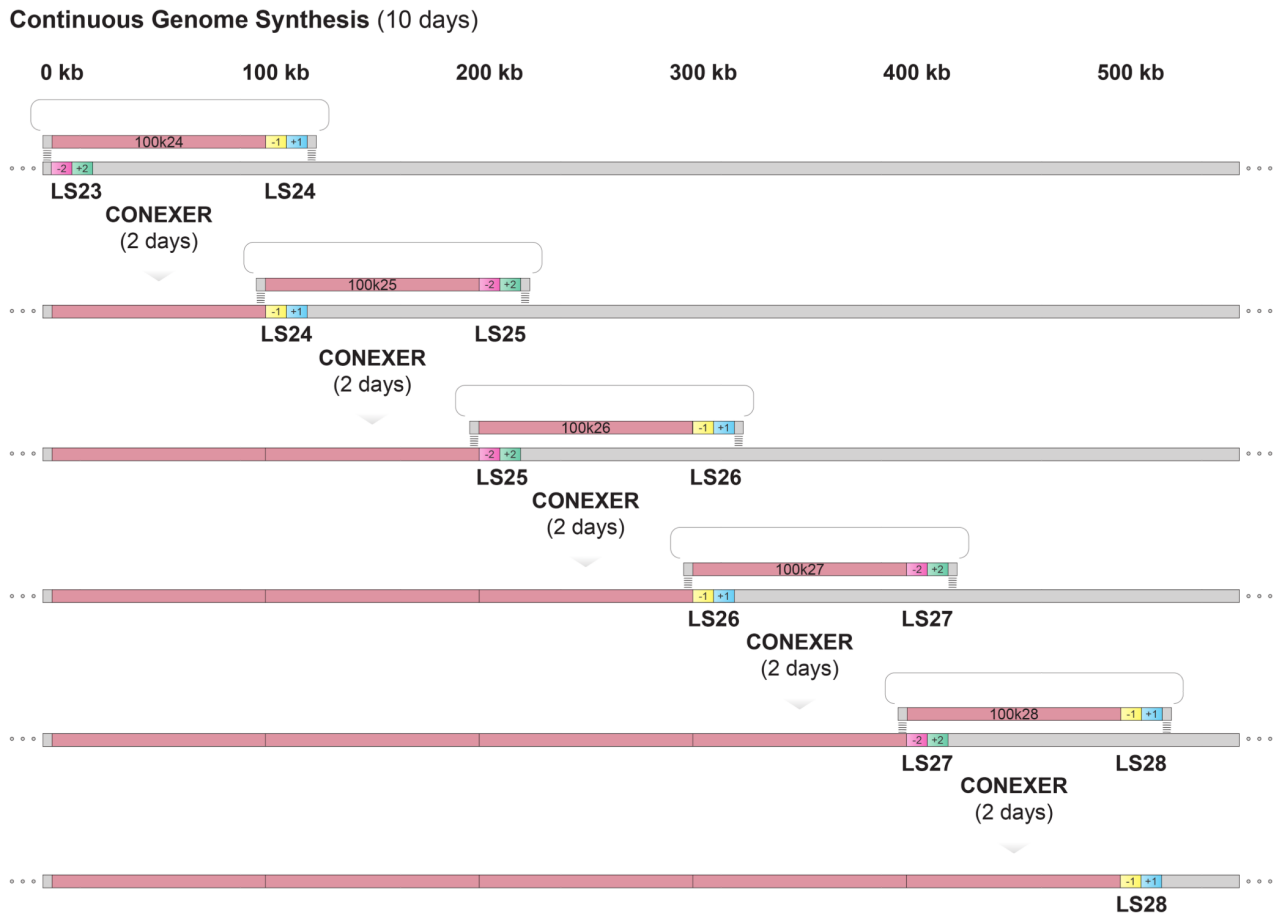
Continuous synthesis of 500 kb of a recoded *E. coli* genome from CONEXER BACs. Continuous genome synthesis via rounds of CONEXER in ∆*recA* recipients. Genomic DNA is depicted in grey and synthetic, recoded DNA in pink. The selectable markers are +1, blue, *kan^R^* (selected for with kanamycin); -1, yellow, *rpsL* (selected against with streptomycin); +2, green, *cat* (selected for with chloramphenicol); -2, pink, *sacB* (selected against with sucrose). Each round of CONEXER replaces approximately 100 kb of the *E. coli* genome with synthetic DNA, and takes two days. Continuous synthesis of a 500 kb synthetic section in the *E.coli* genome was achieved in 10 days.

**Fig. 5:**
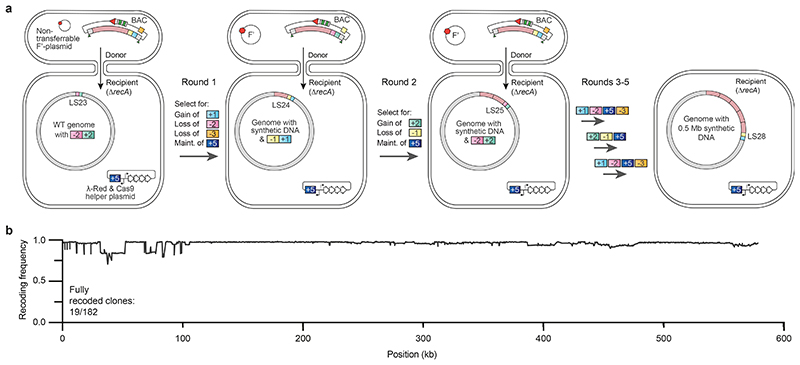
CGS from episomes. **a**, In the first round of CGS we performed CONEXER with a donor cell containing a CONEXER BAC bearing synthetic DNA covering 100k24 and a +1/-1 double selection cassette. The recipient cell contained an appropriately marked genome (-2/+2 at LS23), and had a *recA* deletion. Recipient clones from the selection with the correct set of phenotypes were pooled and acted as the recipient for the next round of CONEXER mediated genome replacement, by virtue of containing +1/-1 at LS24 of their genome. In the next round we performed CONEXER with a donor cell containing a CONEXER BAC bearing synthetic DNA covering 100k25 and a +2/-2 double selection cassette. Recipient clones from the selection with the correct set of phenotypes were pooled and acted as the recipient for the next round of CONEXER mediated genome replacement, by virtue of containing +2/-2 at LS25 of their genome. This process was repeated three more times. After five rounds of CGS, a subset of the resulting cells were expected to have integrated synthetic DNA over the entire 500 kb region. The selectable markers are +1 (blue, *kan^R^*), -1 (yellow, *rpsL*), +2 (green, *cat*), -2 (*pink, sacB*), -3 (orange, pheS*), and +5 (dark blue, *tet^R^*). **b**, The compiled recoding landscape of 182 clones from CGS of 100k24 - 100k28. In total, 19 out of the 182 sequenced clones were fully recoded over the whole 500 kb section of the genome.

We first performed CONEXER, to replace the *E. coli* genome between LS23 and LS24 with synthetic, recoded DNA. We used ∆*recA E. coli* containing the +2/-2 selection cassette at LS23 in its genome and a BAC containing recoded 100k24 with a -1/+1 selection cassette at LS24, and a -3 marker in its backbone (Fig. 5a and Extended Data Fig. 10). We picked colonies from the selection plate and, in parallel with the overnight growth of these colonies in liquid culture, tested for +1, -1, +2, and -2 by examining their growth phenotype on kanamycin, streptomycin, chloramphenicol and sucrose respectively (Extended Data Fig. 9b). The next day, clones with the correct growth phenotypes were pooled, and used as a direct input for the next round of CONEXER (100k25), in which the genomic DNA between LS24 and LS25 was replaced with synthetic DNA (Fig 5a). We selected for the loss of the negative marker from the genome (in the +1/-1 cassette at LS24) and gain of the positive marker associated with the synthetic DNA (in the +2/-2 cassette at LS25). We picked and pooled clones, essentially as described for the previous round of CONEXER, and used the resulting pool as the input for the next round of CONEXER (100k26).

We performed five rounds of CONEXER to replace the 0.5 Mb section of the *E. coli* genome between LS23 and LS28 with synthetic DNA (Fig 5a). The entire process took 10 days. Sequencing revealed that after five rounds of CONEXER 10% of clones (19 out of 182) were fully recoded across the targeted 0.5 Mb region of the genome (Fig 5b). We conclude that we have developed a method for rapid and continuous genome synthesis (CGS) from a set of donor strains, each containing BACs with 100 kb of synthetic DNA.

## Discussion

We have realized a single-step, one-day, universal protocol for introducing at least 100 kb of synthetic DNA from an episome into the *E. coli* genome. We have identified host factor knockouts that minimize crossovers between the host genome and synthetic DNA and developed a CGS method to build a 0.5 Mb section of the *E. coli* genome, from donor cells containing BACs in 10 days. As the methods are parallelizable, it will be possible to build synthetic DNA covering the genome in 7-8 strains, starting from the corresponding, fully characterized, 100 kb BACs in donor strains, in about 10 days. By combining this advance with methods for assembling synthetic DNA in BACs, and rapid and precise methods for compiling 0.5 Mb synthetic genome sections in distinct strains into a single strain^1,7^, we anticipate that our advances may reduce the timescale for the total synthesis of functional *E. coli* genomes to around 2 months (Supplementary Note 1). While constructing organisms with completely synthetic genomes necessarily requires the synthetic genome sequence to be viable, our approach rapidly and precisely identified problematic synthetic sequences that may be fixed using existing, and emerging, methods^1,37^. We anticipate that our approach will enable the construction of many completely synthetic genomes in parallel, enabling genome-level hypotheses to be tested at scale, and the creation of genome libraries for expanding organism function.

We have realized the scarless assembly of episomes bearing large (megabase-scale) regions of the human genome and shown that we can rapidly and precisely edit these episomes in *E. coli*. Future research may leverage the numerous methods for genome editing in *E. coli^38–40^* to further edit large regions of assembled human DNA, much more rapidly than in human, animal or plant cells. Although we have exemplified the principles of our approach through the assembly and modification of natural sequences from human genomic DNA^33^, the approaches may also be used to assemble megabases of DNA from other organisms and to assemble completely synthetic DNA. It may be possible to assemble synthetic chromosomes for diverse microorganisms by BASIS and to transfer the new chromosomes to relevant hosts by, for example, conjugation-based methods; this would provide a route to re-booting diverse microorganisms with synthetic genomes.

We have explicitly shown that we can transfect a 208 kb BASIS BAC into human cells and thereby express the *CFTR* gene. Our methods may be combined with approaches for moving large episomal DNA into human, animal (or plant) cells and for iterative recombination in these cells – to replace natural sequences within chromosomes with synthetic sequence^41–43^ or to insert synthetic sequences into artificial chromosomes. We note that new approaches may be required to move megabases of DNA directly into human or animal cells.

Overall, the ability to rapidly assemble megabase-scale DNA, and the development of CGS, provide key foundations for rapid and scalable genome synthesis.

## Methods

### Strains and plasmids used in this study

We used the following *E. coli* strains in this study: MDS42, MDS42 LowMut ∆*recA* (Scarab Genomics) andDH10b. Strains used for CONEXER and BASIS carry the *rpsL*^K43R^ mutation which confers resistance to streptomycin and enables negative selection against a wild type copy of *rpsL*. We performed yeast assemblies in *Saccharomyces cerevisiae* strain BY4741.

We used the following BACs in this study – 100k09, 100k13, 100k22, 100k24, 100k25, 100k26, 100k27, 100k28 and 100k37a^1^. Each BAC carries ~100 kb of synthetic DNA with a defined synonymous codon compression scheme in which two serine codons (TCG and TCA) and a stop codon (TAG) are replaced through defined recoding rules (TCG to AGC; TCA to AGT; and TAG to TAA).

We used the following positive and negative selection markers in REXER and CONEXER: *rpsL* (-1, streptomycin sensitivity)*, kan^R^* (+1, kanamycin resistance)*, sacB* (-2, conferring sucrose sensitivity), *cat* (+2, chloramphenicol resistance)*, pheS*^T251A_A294G^ (pheS*) (-3, 4-chloro-phenylalanine (4-CP) sensitivity), *hyg^R^* (+3, hygromycin resistance), *gent^R^* (+4, gentamycin resistance), *tet^R^* (+5, tetracycline resistance), and *amp^R^* (+6, ampicillin resistance)^62^. For REXER, we used strains carrying a genomic double selection cassette at the upstream end of the integration site (locus^0^): *rpsL-kan^R^* (+1/-1) for REXER with 100k13 and 100k37a; *sacB-cat* (+2/-2) for REXER with 100k22, 100k24, and 100k28. For CONEXER, we used WT or ∆*recA* strains carrying a genomic double selection cassette at the upstream end of the integration site as recipient cells (locus^0^): *rpsL-kan^R^* (+1/-1) for CONEXER with 100k09, 100k25 and 100k27; *sacB-cat* (+2/-2) for CONEXER with 100k24, 100k26, and 100k28.

We used the helper plasmid pKW20_CDFtet_pAraRedCas9_tracrRNA^4^ (pKW20, NCBI accession MN927219.1) to enable excision and recombination in REXER and CONEXER. pKW20 constitutively expresses a tracrRNA, and Cas9 and λ-red components under the control of an arabinose inducible promoter. Furthermore, we created a derivative plasmid pLF118_Gm_pAraRed(rec)_TracrRNA (pLF118, Supplementary Data 7) without Cas9 to enable λ-red recombination without the expression of Cas9, which was employed to modify BACs for CONEXER (see below). This was done by PCR-amplification of the rest of pKW20 followed by NEBuilder HiFi DNA Assembly.

BACs for the assembly of *CFTR* are based on CONEXER BAC 100k25. The BACs for the assembly of the 1.1 Mb region of the human chromosome 21 are based on BACs from the 32k-human BAC library (BACPAC Genomics)^33^. They were adapted for BASIS using λ-red recombination as described in the Construction of BACs for CONEXER’ section.

For host gene deletions, we used plasmids bearing spacer sequences and pKW20. Spacer plasmids were constructed by restriction-ligation into pMB1 plasmid backbone with single-stranded DNA (ssDNA) oligonucleotides encoding for guides. All spacer sequences are provided in Supplementary Data 1.

For retron-mediated editing we used a plasmid bearing the retron sequence (pFR015_pBAD_retron-RT_araC_bsaI, Supplementary Data 13) and a helper plasmid pFR156_pBAD_CspRecT_MutL-E32K_tracRNA (Supplementary Data 14) encoding CspRecT and *mutL_E32K_* under an arabinose-inducible promoter. The retron plasmid (containing the Ec86 retron scaffold and its cognate reverse transcriptase) was constructed by ligating annealed, phosphorylated ssDNA oligonucleotides into BsaI-digested pFR015.

### Construction of spacer arrays

In this study we performed genomic integration of synthetic DNA from BACs of two different designs (labelled with even and odd numbers, respectively), which required a set of universal spacers each (Universal1 and Universal2). We implemented a third spacer design (Universal3) for BASIS with human BACs as assembly substrates. Note that a series of BACs can be designed so one single universal spacer RNA excises both 5’ and 3’ in all BACs to simplify the method further.

All spacer RNAs for REXER (Supplementary Data 2) were expressed from plasmid pKW3_MB1amp_tracr_Universal1 (Supplementary Data 3) or pKW3_MB1amp_tracr_Universal2 (Supplementary Data 4) (Supplementary Data 2) carrying an *amp^R^* (+6), a tracrRNA, and a spacer array. We constructed each array from overlapping oligonucleotides through two rounds of PCR and prepared the backbone by restriction digestion of pKW3 with AccI and EcoRI^1^. We combined the backbone and each array by NEBuilder HiFi DNA Assembly prior to verification by Sanger sequencing. All of the spacer sequences and oligonucleotide sequences are provided in Supplementary Data 1 and 2.

### Construction of BACs for CONEXER and BASIS

We modified the even BACs for CONEXER by integrating an origin of transfer (*oriT*) sequence to enable conjugative transfer and the universal spacer array (Universal2) on the BAC backbone (Supplementary Data 2). To this end, we coupled the *oriT* and spacer array sequences to the selection marker *amp^R^* (+6). We amplified each sequence by PCR; the plasmid pKW3_MB1amp_tracr_Universal2 served as template for *amp^R^*, F-plasmid RK2^44^ for *oriT*, and pKW3_MB1amp_tracr_Universal2 for the spacer array. We stitched PCR products in two sequential PCRs to create the final *amp^R^*-*oriT*-Universal2 cassette with primers creating 50 bp homology regions to pheS* (-3) and the BAC backbone. We used the Cas9-free helper plasmid pLF118 to initiate λ-red recombination and selected for the integration of the cassette onto the BAC with ampicillin. The complete integration of the cassette was first verified by Sanger sequencing and the successfully modified BAC 100k24 was additionally verified by next-generation sequencing (NGS) to ensure the integrity of the entire synthetic DNA insert. A list of the corresponding oligonucleotide sequences are listed in Supplementary Data 2.

Odd numbered BACs can be modified in an analogous way for CONEXER. The corresponding universal spacer array, Universal1, was amplified from the pKW3_MB1amp_tracr_Universal1 plasmid described above. Corresponding oligonucleotide sequences are listed in Supplementary Data 2.

The odd and even CONEXER BACs provide a simple and rapid basis for integrating synthetic DNA at any point in the *E. coli* genome using the CONEXER protocol. To this end, the BAC backbones may directly be amplified – using the described BACs as templates – for *S. cerevisiae*-mediated assembly of BACs with other synthetic DNA^1,4,45^.

For assembly of the 1.1 Mb target region on chromosome 21, we used BACs from the human genome high-resolution BAC re-arrayed clone set^33^ (32k set; Supplementary Data 2). BACs from this library were adapted for BASIS by integration of an *oriT* sequence, a universal spacer array, a uHR, a double selection cassette (+3/-3, pheS*-hygR for odd BACs; +1/-1, *rpsL-kanR* for even BACs), and a negative selection marker (-1, *rpsL* for odd BACs; -3, pheS* for even BACs). To this end, we cloned plasmids pHBA008_BASIS-components_rK_3-prime (Supplementary Data 15) and pHBA010_BASIS-components_pH_3-prime (Supplementary Data 16) containing all components in the correct orientation by Gibson assembly. These plasmids served as a template for PCR; we amplified the complete sequence to be integrated into the BACs as one linear piece of ~4 kb DNA. We used the Cas9-free helper plasmid pLF118 to initiate λ-red recombination and selected for the integration of the cassette onto the BAC with appropriate antibiotics (+3, 200 μg/mL hygromycin or +1, 50 μg/mL kanamycin) depending on the type of double selection cassette used. The complete integration of the cassette was first verified by genotyping the junctions at both ends of the cassette. Successfully modified BACs were additionally verified by short-read next-generation sequencing (NGS) to ensure the integrity of the entire synthetic DNA insert.

BACs for the assembly of the *CFTR* gene were assembled from DNA fragments in yeast^45^. Fragments were generated via PCR amplification. CONEXER BAC 100k25 was used as a template for the amplification of BAC backbone fragments. Genomic DNA purified from hTERT RPE-1 cells was used as a template for PCR amplification of fragments of the *CFTR* gene which we used for assembly.

To generate donor cells for CONEXER/BASIS/CGS we delivered a non-transferable F’ plasmid to cells containing a sequence verified BAC. For the CONEXER/CGS experiments, the integrity of BACs in donor cells was reverified by conjugating BACs into cells without the F’ plasmid and sequencing using NGS (this enabled high-coverage sequencing of the BAC without the F’ plasmid). This step identified clones, that is, donor cells, containing the intact BAC and F’ plasmid, which were used directly as donors in CONEXER or CGS. Phenotypic verification of the selection markers on BACs is recommended in addition to sequence verification by NGS. Note that strains carrying BACs with negative selection markers do not show sensitivity to chemicals that are normally toxic with these markers, because the episome encoding for the negative selection marker gene will be lost under selective pressure. Negative selection markers on BACs can be verified when maintenance of the BAC is ensured by selection. For example: a strain carrying an episome with a functional +1/-1 (+1, *kanR*, confers resistance to kanamycin; -1, *rpsL*, confers sensitivity to streptomycin) double selection cassette is expected to grow on kanamycin as well as on streptomycin but not on the combination of both.

### REXER

We performed REXER^1,4,45^ in *E. coli* cells containing the helper plasmid pKW20 and a genomic double selection cassette, we transformed the cells with the relevant BAC and plated on LB agar with selection for the helper plasmid (+5, 5 μg/mL tetracycline), selection for the BAC (+2, 20 μg/mL chloramphenicol or +1, 50 μg/mL kanamycin), and suppression of Cas9 and λ-red expression (2% glucose). We inoculated an isolated colony in LB medium with selection for the helper plasmid (+5, 5 μg/mL tetracycline) and the BAC (+2, 20 μg/mL chloramphenicol or +1, 50 μg/mL kanamycin) and incubated the culture overnight at 37 °C with shaking. To render the cell induced and competent, we diluted the overnight culture 1:50 in LB medium with selection for the helper plasmid (+5, 5 μg/mL tetracycline) and the BAC (+2, 20 μg/mL chloramphenicol or +1, 50 μg/mL kanamycin). When cells reached an OD_600_ of around 0.2 (usually after 2 h), we induced expression of λ-red and Cas9 by adding arabinose to a final concentration of 0.5 % (w/v) and continued incubation for one additional hour at 37 °C with shaking. We collected the cells and rendered them electrocompetent^1,45^.

For genomic integration of synthetic DNA by REXER, we transformed the electrocompetent, induced cells with 2 μg of plasmid pKW3_MB1amp_tracr_spacers encoding spacer RNAs. After 1 h of recovery in 4 mL SOB medium with shaking at 37 °C, we transferred the culture to 50 mL LB medium with selection for the helper plasmid (+5, 5 μg/mL tetracycline), the plasmid encoding the spacers (+6, 100 μg/mL ampicillin), the BAC (+2, 20 μg/mL chloramphenicol or +1, 50 μg/mL kanamycin), and continued incubation at 37 °C with shaking for 3 h. We plated the culture on LB agar with selection for the helper plasmid (+5, 5 μg/mL tetracycline), the BAC (+2, 20 μg/mL chloramphenicol or +1, 50 μg/mL kanamycin), and agents selecting against the negative marker on the genome (-1, 200 μg/mL streptomycin or -2, 7.5 % sucrose) as well as the negative marker on the BAC backbone (-1, 200 μg/mL streptomycin or -3, 2.5 mM 4-CP [alternatively: -2, 7.5% sucrose; even steps can also be performed with equivalent markers on BAC backbone and locus^0^]). After overnight incubation at 37 °C, we picked 10-11 colonies and resuspended them in 30 μL water. We assessed each clone by colony PCR for the loss of the upstream genomic double selection cassette (*locus^0^*) and genomic integration of the downstream double selection cassette (*locus^1^*). To assess the correct integration of excised sequences with flap ends, we further verified the first five clones by Sanger sequencing of the colony PCR-products. A list of all oligonucleotide sequences is provided in Supplementary Data 2.

### CONEXER

CONEXER requires preparation of a conjugation competent donor cell and preparation of a recipient cell. The donor cell carries the non-transferable conjugative plasmid and the BAC with the synthetic DNA for genomic integration, an *oriT* sequence and a universal spacer array. The orientation of the *oriT* ensures that the spacer array enters the recipient cell last to minimize the risk of partial excision by premature initiation of Cas9 cleavage in the recipient cell. The recipient cell carries a genomic double selection cassette at *locus^0^*, marking the upstream end of the integration site, and the helper plasmid pKW20 for inducible expression of Cas9 and λ-red components. Odd- and even-numbered BACs can be alternated for replacements of adjacent genomic regions in iterative CONEXER steps, with an alternating selection strategy, essentially as described for REXER and GENESIS^1,4^.

Here, we describe CONEXER with a donor strain carrying an even (or odd) numbered BAC with a 100 kb synthetic DNA insert with *rpsL-kan^R^* (+1/-1) (or *sacB-cat* (+2/-2)) followed by pheS* (-3) (or *rpsL* (-1)) on the BAC backbone; and a recipient strain carrying a genomic *sacB-cat* (+2/-2) (or *rpsL-kan^R^* (+1/-1)) selection cassette at *locus^0^*. We grew the donor strain to saturation overnight in 25 ml LB medium with selection for non-transferable F’ plasmid (50 μg/mL apramycin) and selection for the BAC (+1, 50 μg/mL kanamycin or +2, 20 μg/mL chloramphenicol). We grew the recipient strain to saturation overnight in 25 ml LB medium with selection for the helper plasmid (+5, 5 μg/mL tetracycline), the genomic double selection cassette (+2, 20 μg/mL chloramphenicol or +1, 50 μg/mL kanamycin) and suppression of Cas9 and λ-red expression (2% glucose). We collected the cells from each culture by centrifugation and washed the pellets three times in 1 mL LB medium. After the final wash, we resuspended the pellets in 800 µl LB. We mixed 160 µl of recipient with 640 µl of donor, spotted the mixture onto LB agar plates and, once the spots were dried, incubated the plates at 37 °C for 1 h. After conjugative transfer, we washed cells off the plate and transferred all cells into 250 mL prewarmed LB medium with selection for recipient cells carrying the helper plasmid (+5, 5 μg/mL tetracycline), and the BAC (+1, 50 μg/mL kanamycin or +2, 20 μg/mL chloramphenicol). We induced expression of Cas9 and λ-red (0.5% L-arabinose). After 1.5 h of incubation at 37 °C with shaking we collected cells by centrifugation and immediately transferred them into 250 ml prewarmed LB with selection for recipient cells carrying the helper plasmid (+5, 5 μg/mL tetracycline), and the BAC (+1, 50 μg/mL kanamycin or +2, 20 μg/mL chloramphenicol), and 2% glucose to terminate recombination by supressing expression of Cas9 and λ-red. After another 2.5 h incubation with shaking at 37°C, we centrifuged the culture and resuspended the pellet in 2 mL Milli-Q filtered water. The cell suspension was spread in serial dilutions on LB agar plates with selection for the helper plasmid (+5, 5 μg/mL tetracycline), selection for the integration of the double selection cassette at *locus^1^* (+1, 50 µg/ml kanamycin or +2, 20 μg/mL chloramphenicol), selection for the loss of the double selection cassette at *locus^0^* (-2, 7.5% sucrose or -1, 200 µg/ml streptomycin), and selection for the loss of the BAC backbone (-3, 2.5 mM 4-CP or -1 200 µg/ml streptomycin [not added in addition as the selection marker on the backbone is equivalent to the one at *locus^0^* in this case]). In selection plates without sucrose, we added 2 % glucose to supress Cas9 and λ-red expression.

After overnight incubation at 37 °C, we picked 24-32 colonies and resuspended them in 30 μL water. We assessed each by growth separately on 50 µg/ml kanamycin (presence of +1 marker), 20 μg/mL chloramphenicol (presence of +2 marker), 200 µg/ml streptomycin (presence of -1 marker) and 7.5% sucrose (presence of -2 marker) for loss of the double selection cassette at *locus^0^* and gain of the double selection cassette at *locus^1^*. We selected 5-16 colonies with a verified phenotype for whole-genome sequencing by NGS (Extended Data Fig. 2c). The colony count was determined either by counting colonies manually on a subsection of the plate and linearly scaling to the whole surface or using automated counting on the QPIX420 system (Molecular Devices; settings: debris, ~0.2; axis ratio, ~0.25).

For experiments in *recA*-deletion strains (apart from the initial screen), we grew cells for 2-8 h in 250 ml prewarmed LB with selection for the BAC (+1, 50 μg/mL kanamycin or +2, 20 μg/mL chloramphenicol), and the helper plasmid (+5, 5 μg/mL tetracycline) to enable cells that had received the BAC to expand before the 1.5 h induction of Cas9 and λ-red expression. This increased the number of successful recombinants from CONEXER experiments.

For diagnostic CONEXER experiments we used CONEXER BAC 100k09. The synthetic insert on this BAC contains non-viable recoding events at positions 37,213, 37,227, and 37,251. CONEXER experiments were performed in WT and ∆*recA* genetic background in triplicates. From each CONEXER experiment 14-16 clones were sequenced. For all diagnostic CONEXER experiments a growth time of 2 h before induction of Cas9 and λ-red was used.

### BASIS

BASIS requires preparation of a conjugation competent donor cell and a recipient cell carrying an assembly BAC.

Here, we describe the assembly of the *CFTR* gene in two steps by BASIS. In the first step, we mixed “recipient cells” and “donor cells”. The recipient cells contain the assembly BAC with the first section of the *CFTR* gene followed by a +2/-2 double selection cassette (+2, *cat*(confers growth on chloramphenicol); -2, *sacB*(confers sensitivity to sucrose)) and a plasmid conferring tetracycline resistance (+5, *tet^R^*) that encodes for arabinose-inducible λ-red components and Cas9 protein. The donor cells contain a donor BAC encoding the second section of the *CFTR* gene followed by a +3/-3 double selection cassette (+3, *hyg^R^* (confers growth on hygromycin); -3, pheS* (confers sensitivity to 4-chlorophenylalanine)) and a non-transferable F’ plasmid. The donor BAC contains a -2 marker on the backbone (-2, *sacB* (confers sensitivity to sucrose)). We grew the donor strain to saturation overnight in 25 ml LB medium with selection for non-transferable F’ plasmid (50 μg/mL apramycin) and selection for the BAC (+3, 200 μg/mL hygromycin). We grew the recipient strain to saturation overnight in 25 ml LB medium with selection for the helper plasmid (+5, 5 μg/mL tetracycline), the +2/-2 double selection cassette on the assembly BAC (+2, 20 μg/mL chloramphenicol) and suppression of Cas9 and λ-red expression (2% glucose). We collected the cells from each culture by centrifugation and washed the pellet three times in 1 mL LB medium. After the final wash, we resuspended the pellets in 800 µl LB. We mixed 160 µl of recipient with 640 µl of donor, spotted the mixture onto LB agar plates and, once the spots were dried, incubated the plates at 37 °C for 1 h to facilitate conjugative transfer of the donor BAC to the recipient cell. After conjugative transfer, we washed cells off the plate and transferred them all into 250 mL prewarmed LB medium with selection for recipient cells carrying the helper plasmid (+5, 5 μg/mL tetracycline), and the donor BAC (+3, 200 μg/mL hygromycin), and induced expression of Cas9 and λ-red (0.5% L-arabinose). Spacers expressed from the donor BAC led to the excision of section two of the *CFTR* gene and the adjacent +3/-3 marker from the donor BAC. The ends of the excised linear DNA are homologous to the regions flanking the +2/-2 marker on the assembly BAC. Recombination leads to the replacement of the +2/-2 marker with the linear piece of DNA. After 1.5 h of incubation at 37 °C with shaking we collected cells by centrifugation and immediately transferred them into 250 ml prewarmed LB with selection for recipient cells carrying the helper plasmid (+5, 5 μg/mL tetracycline), the donor BAC (+3, 200 μg/mL hygromycin), and 2% glucose to terminate recombination by supressing expression of Cas9 and λ-red. After another 2.5 h incubation with shaking at 37°C we spun the culture by centrifugation and resuspended the pellet in 2 mL Milli-Q filtered water. The cell suspension was spread in serial dilutions onto LB agar plates with selection for the helper plasmid (+5, 5 μg/mL tetracycline), selection for the integration of the +3/-3 double selection cassette from the donor BAC (+3, 200 μg/mL hygromycin), selection for the loss of the +2/-2 double selection cassette from the assembly BAC (-2, 7.5% sucrose), and selection for the loss of the donor BAC backbone (-2, 7.5% sucrose [not added in addition as the selection marker on the backbone is equivalent to the one on the assembly BAC]).

This procedure results in selection for recipient cells containing an intermediate assembly BAC encoding section one and two of the *CFTR* gene followed by a +3/-3 double selection cassette and a tetracycline resistance conferring plasmid (+5, *tet^R^*) encoding arabinose inducible λ-red components and Cas9. We screened 13 clones from the selection plate by phenotyping and PCR genotyping of the assembly junctions. All 13 clones had the correct phenotype on all four phenotype plates, and four clones had the correct PCR based genotype (the primers are shown Supplementary Data 2). These four clones were further characterized by NGS. A correctly assembled clone (as determined by NGS) was used as the recipient cells for the second step of BASIS.

We mixed these recipient cells with donor cells containing a donor BAC encoding the third section of the *CFTR* gene followed by a +2/-2 double selection cassette (+2, *cat* (confers growth on chloramphenicol); -2, *sacB* (confers sensitivity to sucrose)) and a non-transferable F’ plasmid. The donor BAC contains a -1 marker on the backbone (-1, *rpsL* (confers sensitivity to streptomycin)). We grew the donor strain to saturation overnight in 25 ml LB medium with selection for non-transferable F’plasmid (50 μg/mL apramycin) and selection for the BAC (+2, 20 μg/mL chloramphenicol). We grew the recipient strain to saturation overnight in 25 ml LB medium with selection for the helper plasmid (+5, 5 μg/mL tetracycline), the +2/-2 double selection cassette on the intermediate assembly BAC (+3, 200 μg/mL hygromycin) and suppression of Cas9 and λ-red expression (2% glucose). We collected the cells from each culture by centrifugation and washed the pellet three times in 1 mL LB medium. After the final wash, we resuspended the pellets in 800 µl LB. We mixed 160 µl of recipient with 640 µl of donor cells, spotted the mixture onto LB agar plates and, once the spots were dried, incubated the plates at 37 °C for 1 h to facilitate conjugative transfer of the donor BAC to the recipient cells. After conjugative transfer, we washed the cells off the plate and transferred them into 250 mL prewarmed LB medium with selection for recipient cells carrying the helper plasmid (+5, 5 μg/mL tetracycline), and the BAC (+2, 20 μg/mL chloramphenicol), and induced expression of Cas9 and λ-red (0.5% L-arabinose). Spacers expressed from the donor BAC led to the excision of section three of the *CFTR* gene and the adjacent +2/-2 marker from the donor BAC. The ends of the excised linear DNA are homologous to the regions flanking the +3/-3 marker on the intermediate assembly BAC. Recombination leads to the replacement of the +3/-3 marker with the linear piece of DNA. After 1.5 h of incubation at 37 °C with shaking, we collected cells by centrifugation and immediately transferred them into 250 ml prewarmed LB with selection for recipient cells carrying the helper plasmid (+5, 5 μg/mL tetracycline), and the BAC (+2, 20 μg/mL chloramphenicol), and 2%glucose to terminate recombination by supressing expression of Cas9 and λ-red. After another 2.5 h incubation with shaking at 37°C, we centrifuged the culture and resuspended the pellet in 2 mL Milli-Q filtered water. The resuspended cells were spread in serial dilutions onto LB agar plates with selection for the helper plasmid (+5, 5 μg/mL tetracycline), selection for the integration of the double selection cassette from the donor BAC (+2, 20 μg/mL chloramphenicol), selection for the loss of the double selection cassette from the assembly BAC (-3, 2.5 mM 4-CP), selection for the loss of the donor BAC backbone (-1, 200 μg/mL streptomycin). We added 2 % glucose to suppress Cas9 and λ-red expression. We phenotyped 12 clones for each of the relevant markers (+2, *cat*; -2 *sacB*; +3, *hyg^R^*; -3 pheS*) and characterized clones with the correct set of phenotypes on all four plates (10) by NGS. A correctly assembled final clone was also characterized by short and long-read sequencing. This confirmed that the BASIS assembly (as described in Fig. 3) was error-free with respect to the input BACs.

To assemble the 1.1 Mb target section of human chromosome 21 in a BAC using BASIS, we used a protocol analogous to the *CFTR* assembly. One important difference is that the assembly BAC contained an additional positive selection marker (+4, *gent^R^*, conferring resistance to gentamycin) enabling us to select for the maintenance of the assembly BAC throughout the BASIS process. Furthermore, we concluded that the recovery time of 2.5 h after λ-red induction is not always sufficient. In these cases, we extended the recovery to 3.5 h or 4.5 h.

For the odd steps, the recipient cells contain the assembly BAC with a positive selection marker on the backbone (+4, *gent^R^*, conferring resistance to gentamycin) and a DNA insert followed by a +1/-1 double selection cassette (+1, *kan^R^* (confers growth on kanamycin); -1, *rpsL* (confers sensitivity to streptomycin)) and a plasmid conferring tetracycline resistance (+5, *tet^R^*) that encodes for arabinose-inducible λ-red components and Cas9 protein. The donor cells contain a donor BAC with a DNA insert followed by a +3/-3 double selection cassette (+3, *hyg^R^* (confers growth on hygromycin); -3, pheS* (confers sensitivity to 4-chlorophenylalanine)) and a non-transferable F’ plasmid. The donor BAC contains a -1 marker on the backbone (-1, *rpsL* (confers sensitivity to streptomycin)). We grew the donor strain to saturation overnight in 25 ml LB medium with selection for non-transferable F’ plasmid (50 μg/mL apramycin) and selection for the BAC (+3, 200 μg/mL hygromycin). We grew the recipient strain to saturation overnight in 25 ml LB medium with selection for the helper plasmid (+5, 5 μg/mL tetracycline), the +1/-1 double selection cassette on the assembly BAC (+1, 50 μg/mL kanamycin), the assembly BAC backbone (+4, 10 μg/mL gentamycin) and suppression of Cas9 and λ-red expression (2% glucose). We collected the cells from each culture by centrifugation and washed the pellet three times in 1 mL LB medium. After the final wash, we resuspended the pellets in 800 µl LB. We mixed 160 µl of recipient with 640 µl of donor, spotted the mixture onto LB agar plates and, once the spots were dried, incubated the plates at 37 °C for 1 h to facilitate conjugative transfer of the donor BAC to the recipient cell. After conjugative transfer, we washed cells off the plate and transferred all of them into 250 mL prewarmed LB medium with selection for recipient cells carrying the helper plasmid (+5, 5 μg/mL tetracycline), the assembly BAC (+4, 10 μg/mL gentamycin), and the donor BAC (+3, 200 μg/mL hygromycin) and induced expression of Cas9 and λ-red (0.5% L-arabinose). Spacers expressed from the donor BAC led to the excision of the DNA insert and the adjacent +3/-3 marker from the donor BAC. The ends of the excised linear DNA are homologous to the regions flanking the +1/-1 marker on the assembly BAC. Recombination leads to the replacement of the +1/-1 marker with the linear piece of DNA. After 1.5 h of incubation at 37 °C with shaking, we collected cells by centrifugation and immediately transferred them into 250 ml prewarmed LB with selection for recipient cells carrying the helper plasmid (+5, 5 μg/mL tetracycline), the assembly BAC backbone (+4, 10 μg/mL gentamycin), and the +3/-3 marker from the donor BAC (+3, 200 μg/mL hygromycin), and 2% glucose to terminate recombination by supressing expression of Cas9 and λ-red. After another 2.5-4.5 h incubation with shaking at 37°C, we centrifuged the culture and resuspended the pellet in 2 mL Milli-Q filtered water. The cell suspension was spread in serial dilutions onto LB agar plates with selection for the helper plasmid (+5, 5 μg/mL tetracycline), selection for the assembly BAC backbone (+4, 10 μg/mL gentamycin), selection for the integration of the +3/-3 double selection cassette from the donor BAC (+3, 200 μg/mL hygromycin), selection for the loss of the +1/-1 double selection cassette from the assembly BAC (-1, 200 μg/mL streptomycin), and selection for the loss of the donor BAC backbone (-1, 200 μg/mL streptomycin [not added in addition as the selection marker on the backbone is equivalent to the one on the assembly BAC]). This procedure results in selection for recipient cells containing an intermediate assembly BAC with a positive selection marker on the backbone (+4, *gent^R^*, conferring resistance to gentamycin) and a DNA insert followed by a +3/-3 marker and a tetracycline resistance conferring plasmid (+5, *tet^R^*) encoding arabinose inducible λ-red components and Cas9. These cells are used as the recipient cells for an even step of BASIS.

For the even steps, the recipient cells contain the assembly BAC with a positive selection marker on the backbone (+4, *gent^R^*, conferring resistance to gentamycin) and a DNA insert followed by a +3/-3 double selection cassette (+3, *hyg^R^* (confers growth on hygromycin); -3, pheS* (confers sensitivity to 4-chlorophenylalanine)) and a plasmid conferring tetracycline resistance (+5, *tet^R^*) that encodes for arabinose-inducible λ-red components and Cas9 protein. The donor cells contain a donor BAC with a DNA insert followed by a +1/-1 double selection cassette (+1, *kan^R^* (confers growth on kanamycin); -1, *rpsL* (confers sensitivity to streptomycin)) and a non-transferable F’ plasmid. The donor BAC contains a -3 marker on the backbone (-3, pheS* (confers sensitivity to 4-chlorophenylalanine)). We grew the donor strain to saturation overnight in 25 ml LB medium with selection for non-transferable F’ plasmid (50 μg/mL apramycin) and selection for the BAC (+1, 50 μg/mL kanamycin). We grew the recipient strain to saturation overnight in 25 ml LB medium with selection for the helper plasmid (+5, 5 μg/mL tetracycline), the +3/-3 double selection cassette on the assembly BAC (+3, 200 μg/mL hygromycin), the assembly BAC backbone (+4, 10 μg/mL gentamycin) and suppression of Cas9 and λ-red expression (2% glucose). We collected the cells from each culture by centrifugation and washed the pellet three times in 1 mL LB medium. After the final wash, we resuspended the pellets in 800 µl LB. We mixed 160 µl of recipient with 640 µl of donor, spotted the mixture onto LB agar plates and, once the spots were dried, incubated the plates at 37 °C for 1 h to facilitate conjugative transfer of the donor BAC to the recipient cell. After conjugative transfer, we washed cells off the plate and transferred all of them into 250 mL prewarmed LB medium with selection for recipient cells carrying the helper plasmid (+5, 5 μg/mL tetracycline), the assembly BAC (+4, 10 μg/mL gentamycin), and the donor BAC (+1, 50 μg/mL kanamycin) and induced expression of Cas9 and λ-red (0.5% L-arabinose). Spacers expressed from the donor BAC led to the excision of the DNA insert and the adjacent +1/-1 marker from the donor BAC. The ends of the excised linear DNA are homologous to the regions flanking the +3/-3 marker on the assembly BAC. Recombination leads to the replacement of the +3/-3 marker with the linear piece of DNA. After 1.5 h of incubation at 37 °C with shaking, we collected cells by centrifugation and immediately transferred them into 250 ml prewarmed LB with selection for recipient cells carrying the helper plasmid (+5, 5 μg/mL tetracycline), the assembly BAC backbone (+4, 10 μg/mL gentamycin), and the +1/-1 marker from the donor BAC (+1, 50 μg/mL kanamycin), and 2% glucose to terminate recombination by supressing expression of Cas9 and λ-red. After another 2.5-4.5 h incubation with shaking at 37°C, we centrifuged the culture and resuspended the pellet in 2 mL Milli-Q filtered water. The cell suspension was spread in serial dilutions onto LB agar plates with selection for the helper plasmid (+5, 5 μg/mL tetracycline), selection for the assembly BAC backbone (+4, 10 μg/mL gentamycin), selection for the integration of the +1/-1 double selection cassette from the donor BAC (+1, 50 μg/mL kanamycin), selection for the loss of the +3/-3 double selection cassette from the assembly BAC (-3, 2.5 mM 4-CP), and selection for the loss of the donor BAC backbone (-3, 2.5 mM 4-CP [not added in addition as the selection marker on the backbone is equivalent to the one on the assembly BAC]). This procedure results in selection for recipient cells containing an intermediate assembly BAC with a positive selection marker on the backbone (+4, *gent^R^*, conferring resistance to gentamycin) and a DNA insert followed by a +1/-1 marker and a tetracycline resistance conferring plasmid (+5, *tet^R^*) encoding arabinose inducible λ-red components and Cas9. These cells were used as the recipient cells for an odd step of BASIS.

For BASIS assembly steps 1-7, we identified clones with marker swap at each step of BASIS on the basis of their assembly junction PCR genotype (using the primers sequences provided in Supplementary Data 2) and/or correct set of phenotypes.

In step 1, we genotyped 32 clones; all 11 clones with the correct genotype also had the correct phenotypes. In step 2, we genotyped and phenotyped 3 clones; all of the clones had the correct genotype, two of the clones had the correct phenotypes. In step 3, we genotyped and phenotyped 7 clones; all 7 clones had the correct genotype and phenotypes. In step 4, we phenotyped 32 clones; 29 clones had the correct phenotypes, 22 of these clones were genotyped and 21 had the correct genotype. In step 5, we phenotyped and genotyped 8 clones; all of the clones had the correct genotype and phenotypes. In step 6, we phenotyped 12 clones; and all of the clones had the correct phenotypes. In step 7, we phenotyped 16 clones and all of the clones had the correct phenotypes. For BASIS assembly step 8, we PCR genotyped 7 clones for the presence of the DNA sequence across the BASIS assembly using multiplex PCR (Qiagen Multiplex PCR Kit, according to the manufacturer’s instructions); these PCR reactions reactions amplified regions covering positions 205550-206615 bp, 532200-533701 bp, and 696759-697301 bp, with respect to the first base of the 1.1 Mb assembly. Three of these clones had the correct set of genotypes, and these three clones also had the correct phenotypes. For BASIS assembly step 9, we PCR-genotyped 140 clones for the presence of the DNA sequence across the BASIS assembly using multiplex PCR (Qiagen Multiplex PCR Kit, according to the manufacturer’s instructions); these PCR reactions amplified regions covering positions 205550-206615 bp, 532200-533701 bp, and 696759-697301 bp, with respect to the first base of the 1.1 Mb assembly; 12 of these clones had the correct set of genotypes, and these 12 clones also had the correct phenotypes. Clones that were correct by genotyping and phenotyping (steps 1-5 and 8-9) or phenotyping (steps 6-7) were sequenced, and a correctly assembled clone was used as the input for the next step of BASIS.

A list of all of the oligonucleotide sequences is provided in Supplementary Data 2.

### CFTR transfection

The *CFTR* clone assembled by BASIS and used for transfection was created before the assembly described in Fig. 3. NGS identified an insertion of a 9.5 kb transposon sequence in intron 7 of this *CFTR* clone (we did not observe transposon insertions in any of the other clones that we characterised in assembling *CFTR* or 1.1 Mb of human DNA by BASIS) and two point mutations resulting from PCRs used to generate the fragments used for assembly. The two point mutations in exon 15 (T to A: G930G; A to G: F931S) were corrected by retron-mediated editing. Clones were screened by Sanger sequencing and verified by NGS. We next replaced the endogenous promoter of *CFTR* on this NGS-verified BAC with an EF-1α constitutive promoter. For this purpose, the EF-1α sequence was coupled to an *amp^R^* (+6) resistance gene and amplified as a PCR product (~50 bp of homology flanking the endogenous promoter sequence). λ-red mediated recombination was used to perform the replacement of the promoter. Selection (+6, 100 μg/mL ampicillin) ensured integration of the PCR product. Clones were screened by genotyping PCR and verified by NGS. Moreover, a 3xHA-tag was inserted at the end of exon 17. For this purpose, we first integrated a double selection cassette (+3/-3, pheS*-hygR) at the intended locus on the BAC through λ-red-mediated recombination. Selection (+3, 200 μg/mL hygromycin) ensured integration of the cassette. Clones were screened by genotyping PCR and verified by NGS. We next replaced the double selection cassette with a PCR product containing the 3xHA-tag sequence through λ-red-mediated recombination. Selection (-3, 2.5 mM 4-CP) ensured the replacement of the cassette with the PCR product. Clones were screened by genotyping PCR and verified by NGS. The retron fixing, introduction of the EF-1α promoter and 3xHA-tag are shown in Extended Data Fig. 5; we also subsequently reproduced these steps for the *CFTR* clone as shown in Fig. 3. We removed the transposon sequence in the clone used for transfection by two-step λ-red-mediated recombination: we replaced the transposon sequence with a double-selection cassette (+3/-3, pheS*-hygR), which we then scarlessly removed with a repair oligonucleotide. We grew *E. coli* cells containing the final modified *CFTR* BAC in 750 mL to an OD_600_ of 2-3 and extracted the BAC using the NucleoBond BAC100 kit (Macherey-Nagel) according the manufacturer’s instructions (Maxi).

We transfected the BAC containing a GFP marker and the HA-tagged *CFTR* gene preceded by the EF-1α constitutive promoter through PEI into HEK293 cells (~10ug DNA per 2 million cells). HEK293 cells were purchased from the European Collection of Authenticated Cell Cultures (ECACC), authenticated by ECACC by short tandem repeat DNA profiling, and tested negative for *Mycoplasma* contamination. GFP-positive cells were sorted on the third day after transfection (total cells sorted: *CFTR*-transfected, 1,906,511; un-transfected negative control, 806,185) and their RNA was isolated using the RNEasy kit (Qiagen) and retrotranscribed (ProtoScript II, NEB). The resulting cDNA and the cDNA of untransfected HEK293 cells was used as template for a nested PCR spanning the whole *CFTR* transcript. The resulting amplicon was run on a gel and verified by NGS (see below).

### NGS

BACs and genomic DNA (gDNA) were extracted from overnight cultures of *E. coli* using the QIAprep Spin Miniprep Kit and DNEasy Blood and Tissue Kit (QIAGEN), respectively. Preparation for NGS has been previously described^1,45^. For preparation of many genomes, an automated workflow was implemented with a Biomek FXp (Beckman Coulter) and the DNAdvance kit (Beckman Coulter) as follows: *E. coli* cultures (200-500 µL) were grown overnight in a 1.2 mL 96-well plate, before resuspension in 100 µL lysis solution (96 µL lysis buffer and 4 µL proteinase K) and incubated at 70 ºC for 30 min. Prebinding buffer (Beckman Coulter, 50 µL) was added to the lysate. Subsequently, BBE binding beads (Beckman Coulter, 100 µL) were added to each well and the plate was vortexed (1,000 rpm, 6 min). The beads were magnetized (5 min), and the supernatant removed, and washed three times with 70 % ethanol (170 µL), before eluting the gDNA with Buffer EBA elution buffer (Beckman Coulter, 100 µL). gDNA was then diluted 1:10 in H_2_O and quantified using the Qubit™ dsDNA HS assay kit (Thermo Fischer Scientific) adapted for a connected fluorescence plate reader (Molecular Devices SpectraMAX I3), using a calibration line and 100 µL total volume in a 96-well plate (excitation/emission: 502nm / 532nm). These data were processed onboard and used to direct subsequent dilution of gDNA to 0.25 ng/µL. Finally, we prepared paired-end sequencing libraries using the Nextera XT DNA Library Preparation Kit (Illumina) according to the manufacturer’s protocol but with reduced volumes: input gDNA (0.2-0.25 ng/µL, 2 µL), TD Buffer (3 µL), ATM (2 µL), NT buffer (1.5 µL), indexes (1 µL), NPM (3.5 µL). Index sequences were generated from the ‘Illumina Adapter Sequences’ support document (Nextera DNA indexes, p.16, dated June 2020), purchased from Biomers and used at 10 µM. The libraries were then purified with AMPure XP magnetic beads (Beckman Coulter) according to the manufacturer’s instructions (7:14 bead:reaction vol. ratio), quantified by Qubit (Thermo Fisher Scientific), pooled and denatured according to manufacturer’s instructions. Biomek automation files and associated files are available at GitHub (https://github.com/JWChin-Lab).

Alternatively, we used the NEBNext® Ultra™ II FS DNA Library Prep Kit for Illumina, according to the manufacturer’s instructions. In brief, we used 30 ng of either BAC or genomic DNA in a total volume of 13 µL, performed enzymatic fragmentation for 5 min, and adapter ligation and PCR amplification with 5 cycles. NEBNext® Multiplex Oligos for Illumina were used as index primers.

Libraries were paired-end sequenced on the MiSeq (Illumina, reagent kit v3 (600 cycles)), HiSeq2500 (Illumina, 200-cycle) or NextSeq 2000 (Illumina, P1 reagent kit v3 (300 cycles), P2 reagent kit v3 (100/200 cycles)) system.

### Long-read Oxford Nanopore sequencing

We extracted total DNA (gDNA and episomal DNA) from 2 mL overnight cultures of *E. coli* using the Gentra Puregene Yeast/Bact. Kit (Qiagen). Cell pellets were resuspended in 600 µL cell lysis solution with proteinase K added at 1 mg/mL and lysed for 15 min at 50 ºC. After addition of 3 µL RNAse A solution, the samples were incubated for 30 min at 37 ºC. Then, 200 µL pre-cooled protein precipitation solution was added and incubated for 5 min on ice. We centrifuged the sample for 5 min at 20,000*g*. The supernatant was collected into 600 µL isopropanol and gently mixed. We centrifuged for 1 min at 4 ºC at 2,000*g*. We discarded the supernatant, washed with 600 µL 70% (v/v) ethanol and repeated centrifugation. After discarding the ethanol, the DNA was gently resuspended in 50 µL elution buffer with 0.02% Triton X-100. We quantified the obtained DNA using the Qubit™ dsDNA HS assay kit. We prepared libraries for long-read Oxford Nanopore sequencing using either the Rapid Barcoding Kit (SQK-RBD004) or the Native Barcoding Kit (SQK-NBD112-24) following manufacturer’s instructions. Final long-read libraries were sequenced on the MinION system (MK1b) on R9.4.1 flow cells (FLO-MIN106D).

### Sequencing data analysis

We performed short-read sequencing data analysis for recoding and CGS with a custom Python script (https://github.com/JWChin-Lab) as previously described in detail^1,45^. To generate recoding landscapes across a target genomic region, we used a custom Python script (available at https://github.com/JWChin-Lab) as described in detail previously^1,45^. The out-put is the frequency of recoding at each target codon plotted across the genomic region in question. Fully recoded clones were identified on the basis of the generated recoding landscape.

We assessed assembly fidelity and structural intactness of BASIS constructs using a custom pipeline integrating 160 bp paired-end Illumina short-read sequencing data and Oxford Nanopore long-read sequencing data. We generated a reference haplotype specific to the BAC input material for the assembly region as the individual BACs for the assembly of a 1.1Mb construct from the human BAC library deviate in haplotype from the GRCh38/hg38 human reference genome assembly. To this end, we aligned short-read data for all used input BACs (Supplementary Data 2) to a concatenated reference file comprising of the GRCh38/hg38 human reference genome assembly, the *E. coli* genome (NCBI accession AP012306.1), and helper plasmids pKW20 and pLF118 with bwa mem (v0.7.17; -M -7)^46^. The alignment files were indexed and filtered using Samtools (v1.16.1)^47^ for unique mapping and pairing (view -q 10 -F 1284 -f 0x02). We computed coverage using deeptools (v3.5.1)^48^ bamCoverage with the bin size set to 50 nucleotides. We performed base quality score recalibration (BQSR) using GATK (v4.3.0)^49^. Mutations of BAC input sequences with respect to GRCh38/hg38 were identified using HaplotypeCaller and filtered using variant quality score recalibration (VQSR). We generated an alternative reference genome (BAC-corrected reference) on the basis of the BAC input sequences using FastaAlternateReferenceMaker. We aligned all BAC and BASIS construct short-read sequencing data against the BAC-corrected reference using bwa and processed the alignment data as follows: alignments were sorted (Samtools sort -@ 4 -m 2G), indexed (Samtools index), multiple alignments were removed (Samtools view -h), and filtered for paired reads (Samtools view q 10 -F 1284 -f 0x02). Duplicates were marked (MarkDuplicates) and read groups were added (AddOrReplaceReadgroups). To identify short variants, we performed candidate short somatic variant calling using Mutect2, calling variants in BASIS assemblies (as tumour sample) against input BAC sequences (matched normal). We filtered the short variants using FilterMutectCalls. The resulting variants were then manually filtered for position (chromosome 21: 32545200-33615150) and allele frequency in BASIS samples (AF >=0.1 in BASIS samples). Moreover, human BAC-library-based BASIS assemblies were processed for germline variant calling (HaplotypeCaller) directly against the BAC-corrected reference. We intersected the resulting short variants called for all input BACs and short variants called for BASIS assemblies, only retaining short variants that were exclusively called for BASIS assemblies. We manually verified and the curated results for short variant calling, classifying variants into three categories: true positive variants (not present in input BAC sequence but present in the BASIS assembly), ambiguous variants (variants for which sequencing data were ambiguous but suggest the presence of a called variant both in the input BAC and the BASIS assembly) and false-positive variants (flagged in the variant calling process but unambiguously present in input BAC sequence as well as final BASIS construct) (Supplementary Data 18 and 19).

We used long-read sequencing data for all input BACs for the BASIS construct at 1.1Mb to verify the structural intactness of the assembled episomal construct. Long-read data were basecalled using guppy-basecaller, demultiplexed using guppy-barcoder and aligned to the *CFTR* BAC reference or the BAC-corrected reference with minimap2. We screened for structural variants both in input BACs and the final assembly using Sniffles2^50^. Similar to short variants, we manually validated structural variants called with Sniffles2 and considered structural variants in BASIS assemblies to be true variants only if they were not present in the respective input BAC (Supplementary Data 18 and 19).

To visualize sequencing traces for the assembly of the 1.1Mb target region, we manually corrected structural variants > 80 bp which were present both in input BACs and the final 1.1Mb BASIS assembly (and therefore classed as false positive; Supplementary Data 19). We aligned BASIS short-read sequencing data to this shortened corrected BAC reference file spanning the 1.1 Mb target region using bwa mem and processed alignment files as described above. We extracted coverage data for all BASIS constructs using Samtools (depth -a). Coverage depth was plotted in 250 bp windows.

We validated the assembly of the *CFTR* BAC as described for the 1.1 Mb assembly, including somatic mutation calling. Furthermore, we manually identified all variants in the final *CFTR* BAC with respect to the *CFTR* sequence (derived from GRCh38/hg38) and scored whether these variants were also present in the input BACs. We did not detect any true-positive variants that were present in the final *CFTR* BAC but not in the input BAC (Supplementary Data 11). For visualization purposes, short-read sequencing data were aligned against the *CFTR* BAC reference (BAC components and *CFTR* sequence were derived from GRCh/hg38), in which coverage depth is plotted in 250 bp windows.

### Genomic features analysis

We analysed the genomic features of the 1.1 Mb target region with respect to the whole genome. To this end, short tandem repeats (STR) and structural feature coordinates were recovered from the non-B DB (https://nonb-abcc.ncifcrf.gov/apps/site/default)^51^. Other features were compiled from the RepeatMasker and regulation UCSC annotation tracks. To compare the distribution of genomic features within BASIS assemblies with the rest of the human genome, we computed fractions of 1 Mb non-overlapping tiles of the genome covered by each feature and compared their distributions to the 1.1 Mb target region.

### Strain generation of host-factor knockouts

For gene knockout using CRISPR/Cas9-mediated cleavage and λ-red recombineering, we adapted a previously described procedure^38^. We cloned spacer plasmids bearing spacer sequences by restriction-ligation into the pSP43_pKW3spec(rec)_SapI_insert_gRNA (Supplementary Data 20) backbone with ssDNA oligonucleotides encoding for guides. In brief, we phosphorylated ssDNA oligonucleotides with T4 PNK (NEB), annealed and ligated with the pSP43 backbone. We transformed the obtained plasmids into *E. coli* cells and verified the sequence using Sanger sequencing. Host-factor single knockouts were performed in *E. coli* cells with a *sacB-cat* double selection cassette integrated at LS23 bearing the helper plasmid pKW20. We grew up cultures in LB to an OD_600_ of 0.2 and then added l-arabinose (0.5 %) to induce Cas9 and λ-red. After 1.5 h of arabinose induction, cells were collected and rendered electrocompetent by washing three times with 50 mL ice-cold 20 % (w/v) glycerol in Milli-Q water. For CRISPR/Cas9-mediated cleavage, a further helper plasmid expressing the target-specific spacer sequence (conferring spectinomycin resistance) was co-electroporated with a repair ssDNA oligonucleotide introducing two stop-codons and a frameshift mutation into the target gene. The cultures were recovered after electroporation in 1 ml SOB for 1 h at 37 °C and then plated onto selective LB agar plates (75 µg/mL spectinomycin, 20 µg/mL chloramphenicol, and 0.5 % L-arabinose for continued Cas9 activity). The next day, we picked colonies from the selective plates and amplified the targeted gene region by colony PCR. Deletions were confirmed by Sanger sequencing. Subsequently, deletion strains were cured of helper plasmids (pHFXX derived from pSP43 with *spec^R^*) by repeated passaging. Curing was confirmed by phenotyping. For *recA*, a two-step λ-red protocol was used in which, in a first recombination, a double-selection cassette (+3/-3) was used to replace the gene of interest. In a second recombination, the double-selection cassette was removed for whole gene deletion.

### Statistical analysis

To evaluate whether deletion of host genes had a significant effect on the full recoding frequency in CONEXER experiments in section 100k24 (Fig. 4), we performed a one-way analysis of variance (in Prism 9) comparing each condition with the WT. To correct for multiple hypotheses, we applied the conservative Bonferroni-correction.

To evaluate the statistical significance of the change in the diagnostic resolution (Extended Data Fig. 8), we performed a two-sided unpaired t-test comparing the *∆recA* and WT conditions (in Prism 9).

### Retron-mediated gene editing

To correct two adjacent single nucleotide mutations in the *CFTR* gene, we adapted a procedure for retron-mediated gene editing^52^. In brief, we cloned a retron plasmid designed to target the lagging strand of the *CFTR* gene (TGATTAGAGTATGCACCAGTGGTAGACCTCTGAAGAATCCCATAGCAAGCAAAGTGTCGGCTACTCCCACGTA). We co-transformed this retron plasmid with pFR156, which contains a gene encoding CspRecT and *mutL*_E32K_, into *E. coli* cells containing the BASIS BAC carrying the *CFTR* gene. An overnight culture of a co-transformant was then diluted 1:100 into fresh LB medium containing 20 µg/mL chloramphenicol, 75 µg/mL spectinomycin, and 50 µg/mL gentamicin. After 1 h at 37 °C with shaking, editing was induced through addition of 0.2 % arabinose and incubated for another 24 h. The cultures were then plated out and colonies were screened by Sanger sequencing of colony PCR products before restreaking and confirmation by NGS.

### CGS

For CGS, CONEXER 100k24 was first performed in *E. coli* ∆*recA* with a *sacB-cat* (+2/-2) double selection cassette at LS23. The next day, 40 clones were picked from the selection plate and grown up individually. We assessed each clone by phenotyping on sucrose, chloramphenicol, streptomycin, and kanamycin (Extended Data Fig. 9) for the loss of the *sacB-cat* (+2/-2) cassette at LS23 and integration of the *rpsL-kan^R^* (+1/-1) cassette at LS24. Clones with the correct set of phenotypes (39) were subsequently pooled at equal ratios to a total volume of 25 mL. This pool of cells was used as the recipient culture for CONEXER 100k25. In total, 96 clones were picked from the selection plate and grown up individually. Again, we assessed each clone by phenotyping for the loss of the *rpsL-kan^R^* (+1/-1) cassette at LS24 and the integration of the *sacB-cat* (+2/-2) cassette at LS25. Clones with the correct set of phenotypes (72) were subsequently pooled at equal ratios to a total volume of 25 mL. This pool of cells was used as the recipient culture for CONEXER 100k26. In total, 96 clones were picked from the selection plate and grown up individually. We then assessed each clone by phenotyping for the loss of the *sacB-cat* (+2/-2) cassette at LS25 and the integration of the *rpsL-kan^R^* (+1/-1) cassette at LS26. Clones with the correct set of phenotypes (53) were subsequently pooled at equal ratios to a total volume of 25 mL. This pool of cells was used as the recipient culture for CONEXER 100k27. The next day, 96 clones were picked from the selection plate and grown up individually. We then assessed each clone by phenotyping for the loss of the *rpsL-kan^R^* (+1/-1) cassette at LS26 and the integration of the *sacB-cat* (+2/-2) cassette at LS27. Clones with the correct set of phenotypes (77) were subsequently pooled at equal ratios to a total volume of 25 mL. This pool of cells was used as the recipient culture for CONEXER 100k28. The next day, 288 clones were picked from the selection plate and grown up individually. We then assessed each clone by phenotyping for the loss of the *sacB-cat* (+2/-2) cassette at LS27 and the integration of the *rpsL-kan^R^* (+1/-1) cassette at LS28. Out of all the clones with the correct set of phenotypes (284) 182 were sequenced using NGS.

To calculate the expected frequency of fully recoded clones in CGS, we multiplied the experimentally determined frequency of fully recoded clones for each step of CONEXER.

## Extended Data

## Supplementary Material

Supplementary Data 1

Supplementary Data 2

Supplementary Data 3

Supplementary Data 4

Supplementary Data 5

Supplementary Data 6

Supplementary Data 7

Supplementary Data 8

Supplementary Data 9

Supplementary Data 10

Supplementary Data 11

Supplementary Data 12

Supplementary Data 13

Supplementary Data 14

Supplementary Data 15

Supplementary Data 16

Supplementary Data 17

Supplementary Data 18

Supplementary Data 19

Supplementary Data 20

Supplementary Information Guide

Supplementary notes; Supplementary Figures

## Data Availability

The sequences and design details used in this study are available in the Supplementary Data. Supplementary Data 1 provides all spacer sequences used in CONEXER and BASIS experiments. Supplementary Data 2 lists all nucleotide sequences of oligonucleotides, plasmids and BACs used in this study. Supplementary Data 3 provides the GenBank file of spacer plasmid pKW3 to express universal spacer set 1. Supplementary Data 4 provides the GenBank file of spacer plasmid pKW3 to express universal spacer set 2. Supplementary Data 5 provides the GenBank file of a general CONEXER BAC design with spacer sequences of universal spacer set 1. Supplementary Data 6 provides the GenBank file of a general CONEXER BAC design with spacer sequences of universal spacer set 2. Supplementary Data 7 provides the GenBank file of plasmid pLF118 for λ-red recombineering. Supplementary Data 8 provides the GenBank file of *CFTR* BAC01. Supplementary Data 9 provides the GenBank file of *CFTR* BAC02. Supplementary Data 10 provides the GenBank file of *CFTR* BAC03. Supplementary Data 11 provides detailed results for sequence verification, listing all variants and their classification as called for the final *CFTR* assembly. Supplementary Data 12 lists all raw sequencing data, including their NCBI SRA accession numbers, as deposited at **BioProject (PRJNA962525)**. Supplementary Data 13 provides the GenBank file of plasmid pFR015 used for retron-editing. Supplementary Data 14 provides the GenBank file of helper plasmid pFR156 for retron-editing. Supplementary Data 15 provides the GenBank file of plasmid pHBA008 which served as template to amplify BASIS components for human BAC adaptation. Supplementary Data 16 provides the GenBank file of plasmid pHBA010 which served as template to amplify BASIS components for human BAC adaptation. Supplementary Data 17 provides the GenBank file of initial assembly acceptor plasmid pHBA031 for the 1.1 Mb BASIS assembly. Supplementary Data 18 provides detailed results for sequence verification, listing all true positive variants as called for the final 1.1 Mb BASIS assembly. Supplementary Data 19 provides detailed results for sequence verification, listing all variants as called for the final 1.1 Mb BASIS assembly and categorised as either likely false positive or false positive. Supplementary Data 20 provides the GenBank file of plasmid pSP43 with spacer sequences for gene knock-out by CRISPR/Cas9-mediated cleavage and λ-red recombineering. All other datasets generated and/or analysed in this study are available from the corresponding author upon reasonable request. All materials (Supplementary Data 3, 4, 7 - 10, 13 – 17 and 20) from this study are available from the corresponding author upon reasonable request.

## Code availability

Code used for generating the recoding landscape (https://github.com/JWChin-Lab/recoding-landscape-generator); for sequence data alignment and analysis (https://github.com/JWChin-Lab/NGS-analysis); for analysis of BASIS constructs (https://github.com/JWChin-Lab/BASIS-sequence-analysis); for automated liquid handling for next generation sequencing library preparation (https://github.com/JWChin-Lab/NGS-sample-prep) is available at GitHub.
