# Supplementary material for: Continuous synthesis of E. coli genome sections and Mb-scale human DNA assembly
Source: Nature. Author manuscript; Available in PMC 2023 Jul 20. (PMC7614783; doi:10.1038/s41586-023-06268-1)
Supplement: Supplementary Information Guide [file EMS177430-supplement-Supplementary_Information_Guide.docx]

**SI Guide**

**Continuous synthesis of *E. coli* genome sections and Mbp human DNA assembly**

Jérôme F. Zürcher^1,†^, Askar A. Kleefeldt^1,†^, Louise F. H. Funke^1,3,†^, Jakob Birnbaum^1,†^, Julius Fredens^1,4,†^, Simona Grazioli^1^, Kim C. Liu^1^, Martin Spinck^1^, Gianluca Petris^1,2^, Pierre Murat^1^, Fabian B.H. Rehm^1^, Julian E. Sale^1^ & Jason W. Chin^1,*^

^1^Medical Research Council Laboratory of Molecular Biology, Francis Crick Avenue, Cambridge, England, UK

^2^Wellcome Sanger Institute, Wellcome Trust Genome Campus, Hinxton, Saffron Walden CB10 1RQ, England, UK

^3^*Present address:* Department of Biomedical Engineering, National University of Singapore, Singapore

^4^*Present address:* Synthetic Biology for Clinical and Technological Innovation, Department of Biochemistry, National University of Singapore, Singapore

**Table of Contents**

| Supplementary Notes; Supplementary Figures | 3 |
| --- | --- |
| Supplementary Data 1-20 | 3-5 |

**Supplementary Notes; Supplementary Figures**

Contains Supplementary Notes 1 and 2 as well as Supplementary Figures 1 to 6.

**Supplementary Data 1 – Table of spacer sequences**

Nucleotide sequences used for spacers in CONEXER and BASIS experiments.

**Supplementary Data 2 – Table of oligos, plasmids, and BACs**

Nucleotide sequences for oligonucleotides, description of plasmids and BACs.

**Supplementary Data 3 – pKW3_MB1amp_tracr_Universal1**

This Genbank file provides the nucleotide sequence for an annotated vector used for REXER experiments to express spacer sequence set Universal 1.

**Supplementary Data 4 – pKW3_MB1amp_tracr_Universal2**

This Genbank file provides the nucleotide sequence for an annotated vector used for REXER experiments to express spacer sequence set Universal 2.

**Supplementary Data 5 – CONEXER_BAC_Universal1**

This Genbank file provides the nucleotide sequence for an annotated BAC vector backbone for CONEXER, providing overview on CONEXER BAC architecture. The synthetic insert is annotated with a placeholder. The vector encodes spacer set Universal 1.

**Supplementary Data 6 – CONEXER_BAC_Universal2**

This Genbank file provides the nucleotide sequence for an annotated BAC vector backbone for CONEXER, providing overview on CONEXER BAC architecture. The synthetic insert is annotated with a placeholder. The vector encodes spacer set Universal 2.

**Supplementary Data 7 – pLF118_Gm_pAraRed(rec)_TracrRNA**

This Genbank file provides the nucleotide sequence for an annotated vector, derived from pKW20_CDFtet_pAraRedCas9_tracrRNA (NCBI accession: MN927219), lacking Cas9.

**Supplementary Data 8 – CFTR_BAC01**

This Genbank file provides the nucleotide sequence for an annotated BAC vector containing section III of the CFTR gene and components for BASIS assembly. The final BAC with the full-length CFTR gene was assembled from CFTR_BAC01 with CFTR_BAC02 and CFTR_BAC03.

**Supplementary Data 9 – CFTR_BAC02**

This Genbank file provides the nucleotide sequence for an annotated BAC vector containing section II of the CFTR gene and components for BASIS assembly.

**Supplementary Data 10 – CFTR_BAC03**

This Genbank file provides the nucleotide sequence for an annotated BAC vector containing section I of the CFTR gene and components for BASIS assembly.

**Supplementary Data 11 – Table of CFTR variants**

Table of identified CFTR variants of the final full CFTR assembly, with list of all variants that are called in the analysis pipeline that are true positive variants (0), ambiguous variants (1) and false positive variants (153).

**Supplementary Data 12 – Table of raw sequencing data and accession numbers**

List and description of raw sequencing data including accession numbers (BioSample and SRA accession) as submitted to NCBI SRA in BioProject **PRJNA962525.**

**Supplementary Data 13 – pFR015_pBAD_retron-RT_araC_bsaI**

This Genbank file provides the nucleotide sequence for an annotated vector for retron editing with the retron operon under control of an arabinose-inducible promoter.

**Supplementary Data 14 – pFR156_pBAD_CspRecT_MutL-E32K_tracRNA**

This Genbank file provides the nucleotide sequence for an annotated vector for retron editing, encoding for a mutL variant and recT.

**Supplementary Data 15 – pHBA008_BASIS-components_rK_3-prime**

This Genbank file provides the nucleotide sequence for an annotated cloning vector with all relevant components for BASIS assembly. The vector bears a *rpsl-Kan^R^* cassette. The vector serves as PCR template for adaptation human library BACs for BASIS experiments.

**Supplementary Data 16 – pHBA010_BASIS-components_pH_3-prime**

This Genbank file provides the nucleotide sequence for an annotated cloning vector with all relevant components for BASIS assembly. The vector bears a *pheS*-Hyg^R^* cassette. The vector serves as PCR template for adaptation human library BACs for BASIS experiments.

**Supplementary Data 17 – pHBA031_BASIS-LS-rK-BAC266-PH-3**

This Genbank file provides the nucleotide sequence for an annotated vector to serve as empty assembly BAC for BASIS. The vector harbours a 60 bp homology with the first adapted human library BAC, BAC266-pH, used for assembly of the 1.1 Mb human DNA construct.

**Supplementary Data 18 – Table of BASIS 1.1 Mbp true positive variants**

Table of variants in final 1.1 Mbp BASIS assembly that were called and identified as true positive variants (5).

**Supplementary Data 19 – Table of BASIS 1.1 Mbp ambiguous and false positive variants**

Table of variants in final 1.1 Mbp BASIS assembly that were called and identified as ambiguous variants that are likely false positive (8) or confidently identified as false positive (39). The table also includes a list of structural variants that were called during the analysis, including information on whether the structural variant was identified by manual analysis (1) or detected with Sniffles2 (4). All structural variants are false positive (5).

**Supplementary Data 20 – pSP43_pKW3spec(rec)_SapI_insert_gRNA**

This Genbank file provides the nucleotide sequence for an annotated vector for gRNA expression for experiments for gene knock-out by CRISPR/Cas9-mediated cleavage and λ-red recombineering.
