# Supplementary material for: Continuous synthesis of E. coli genome sections and Mb-scale human DNA assembly
Source: Nature. Author manuscript; Available in PMC 2023 Jul 20. (PMC7614783; doi:10.1038/s41586-023-06268-1)
Supplement: Supplementary notes; Supplementary Figures [file EMS177430-supplement-Supplementary_notes__Supplementary_Figures.pdf]

Supplementary Information

Supplementary Notes; Supplementary Figures

Continuous synthesis of *E. coli* genome sections and Mbp human DNA assembly

Jérôme F. Zürcher[1,†], Askar A. Kleefeldt[1,†], Louise F. H. Funke[1,3,†], Jakob Birnbaum[1,†], Julius Fredens[1,4,†], Simona Grazioli[1], Kim C. Liu[1], Martin Spinck[1], Gianluca Petris[1,2], Pierre Murat[1], Fabian B.H. Rehm[1], Julian E. Sale[1] & Jason W. Chin[1,*]

[1]Medical Research Council Laboratory of Molecular Biology, Francis Crick Avenue, Cambridge, England, UK

[2]Wellcome Sanger Institute, Wellcome Trust Genome Campus, Hinxton, Saffron Walden CB10 1RQ, England, UK

[3]*Present address:* Department of Biomedical Engineering, National University of Singapore, Singapore

[4]*Present address:* Synthetic Biology for Clinical and Technological Innovation, Department of Biochemistry, National University of Singapore, Singapore

[†]These authors contributed equally.

[*]Correspondence: chin@mrc-lmb.cam.ac.uk

Table of Contents

Supplementary Note 1 3

Supplementary Note 2 6

Supplementary Figures 1-6 8

Supplementary Note 1

Steps and timelines in whole-genome synthesis

This work takes BACs containing approximately 100kbp of DNA as the starting materials for genome synthesis. There are several aspects to genome synthesis and design.

First, we design a genome, this is a computational task that can take anything from a few hours to a few months depending on the complexity of design. As genome design is in its infancy (two functional, cellular genomes[1-3] and several yeast chromosomes[4,5] have been synthesized) we cannot yet predict whether a synthetic genome design will work, and to what extent new genome designs will be tolerated. The genome design for Syn61 was based in empirically determined recoding rules which were selected based on a REXER experiments with defined recoding schemes on a 20kbp region of the genome[6].

Encouragingly, the Syn61 design worked with very few exceptions (4 problematic regions) distributed across three 100kbp sections[2].

Next, we need to test the design. There are several limiting strategies for testing genome designs. One strategy is to build the entire synthetic genome design and move it into the host of interest and use the synthetic genome to replace the natural genome; a challenge with this strategy (as used for mycoplasma[3,7]) is that a single deleterious change in the synthetic genome can render the transfer impossible. A second strategy is to iteratively replace stretches of the genome with the synthetic DNA corresponding to the genome design; this strategy has the advantage that it can localize problematic regions, which can then be fixed, but the rate of genome synthesis is limited to the rate at which we can fix each problematic region in series. A third strategy is to replace stretches of the genome with synthetic DNA in parallel strains and to identify problematic regions in parallel; this strategy has the advantage that we identify all the problems in distinct stretches of the design at once and can then focus on fixing all of them at once.

To create Syn61 we used a combination of the second and third approach[2]. We tested all the synthetic stretches through parallel REXERs to rapidly identify problematic regions and we iteratively built 0.5Mbp sections in 100kbp steps; this allowed us to identify the problems in our design as quickly as possible and to generate maximally recoded 0.5Mbp sections. We were then able to work in parallel on fixing the design in the problematic stretches and building a genome in which as many stetches as possible are composed of synthetic DNA.

We showed (**Extended Data Fig. 8**) that the CONEXER conditions used for CGS can define synthetic sequences in individual stretches with a precision that is at least as good as that achieved by REXER. Therefore, parallel CONEXERs on individual stretches can be used to define the problematic regions from the compiled recoding landscapes, prior to fixing the genome design and synthesis of the genome. Alternatively, Continuous genome synthesis can be run for five steps to generate 0.5Mbp sections. The stretches that contain synthetic sequences that are not tolerated (and the sequences within stretches that are not tolerated) will be directly defined in the compiled recoding landscape derived from sequencing the genomes of clones following CGS. These sequences can then be targeted for fixing in individual maximally recoded clones.

A variety of methods have been reported for refining problematic regions and fixing synthetic genome designs[4,6,8]. However, it is not currently possible to estimate for any design the extent to which the design will need to be corrected or the type of corrections that will be required; as a result it is not possible to estimate the amount of time it will take to correct the original design for any synthetic genome. Because we do not know in advance the extent to which any genome design will be tolerated we cannot predict the timescale for completing the fixing of synthetic sequences that are not tolerated.

In this work we have shown that we can synthesize 0.5Mbp sections of the *E. coli* genome from 100kbp BACs in 10 days (**Fig. 5**). We have previously shown that 0.5Mbp sections can be compiled into the single Syn61 genome through a conjugation-based approach[2], other conjugation-based approaches have also been described[9], and we have described genome fission-fusion strategies for compiling genomes in *E. coli*[10]. In our approach to compiling genomes into a single strain each conjugation takes about five days (this includes modifying strains for conjugation, transforming a non-transferable F' plasmid into the conjugative donor, performing conjugative transfer and selecting the cell with the compiled genome. All eight 0.5Mbp sections created by REXER/CGS can be adapted and conjugated in parallel, the resulting four 1Mbp sections can be adapted and conjugated in parallel and the final two 2Mbp sections can be adapted and conjugated in parallel. Therefore, the total time for convergently compiling the 4Mbp genome from 0.5Mbp sections is about 15 days plus a few days for sequencing of intermediates, giving a total of around 20 days.

A variety of methods can be used to make the 100kbp BACs used as inputs for continuous genome synthesis. Key intermediates in the synthesis of 100kbp BACs are commonly vectors bearing approximately 5-10kbp of synthetic DNA[1,2]. These can be assembled, within a week, into sequenced BACs by homologous recombination in yeast or by in vitro assembly methods such as Gibson assembly[11-13]. The choice of starting materials for making these 10kbp BACs will depend on several factors including cost, speed, and how much of the supply chain it is desirable to own. For example, 10kbp can be purchased directly from commercial suppliers, but in our experience the wait is currently months. Alternatively, the entire process from oligo design to having vectors from which to assemble 100kbp stetches can be completed by enzymatic in vitro assembly methods in a week[7] and therefore the process from oligonucleotides to sequenced 100kbp BACs completed in 2 weeks[7]; it is likely that the drive to increase the length, speed, and scale of oligo synthesis will further accelerate these processes.

Thus, for a viable genome design the timeline from designing oligonucleotides to complete synthetic genome is approximately 7 weeks, this is composed of 2 weeks for BAC creation, ten days for CGS, and 20 days for genome compilation. Automation may allow the scaling of these approaches to the synthesis of many genomes in parallel. While challenge remain in the design of viable genomes and the fixing of disallowed synthetic sequences, the ability to synthesize genomes rapidly and in parallel should lead to a greater understanding of how to design functional genomes that are even more radically altered.

1 Gibson, D. G. *et al.* Complete chemical synthesis, assembly, and cloning of a Mycoplasma genitalium genome. *Science* **319**, 1215-1220, doi:10.1126/science.1151721 (2008).
2 Fredens, J. *et al.* Total synthesis of Escherichia coli with a recoded genome. *Nature* **569**, 514-518, doi:10.1038/s41586-019-1192-5 (2019).
3 Gibson, D. G. *et al.* Creation of a bacterial cell controlled by a chemically synthesized genome. *Science* **329**, 52-56, doi:10.1126/science.1190719 (2010).

4 Annaluru, N. *et al.* Total Synthesis of a Functional Designer Eukaryotic Chromosome. *Science* **344**, 55-58, doi:10.1126/science.1249252 (2014).
5 Richardson, S. M. *et al.* Design of a synthetic yeast genome. *Science* **355**, 1040-1044, doi:10.1126/science.aaf4557 (2017).
6 Wang, K. H. *et al.* Defining synonymous codon compression schemes by genome recoding. *Nature* **539**, 59-+, doi:10.1038/nature20124 (2016).
7 Hutchison, C. A., 3rd *et al.* Design and synthesis of a minimal bacterial genome. *Science* **351**, aad6253, doi:10.1126/science.aad6253 (2016).
8 Napolitano, M. G. *et al.* Emergent rules for codon choice elucidated by editing rare arginine codons in Escherichia coli. *Proceedings of the National Academy of Sciences of the United States of America* **113**, E5588-E5597, doi:10.1073/pnas.1605856113 (2016).
9 Ma, N. J., Moonan, D. W. & Isaacs, F. J. Precise manipulation of bacterial chromosomes by conjugative assembly genome engineering. *Nat Protoc* **9**, 2285-2300, doi:10.1038/nprot.2014.081 (2014).
10 Wang, K., de la Torre, D., Robertson, W. E. & Chin, J. W. Programmed chromosome fission and fusion enable precise large-scale genome rearrangement and assembly. *Science* **365**, 922-926, doi:10.1126/science.aay0737 (2019).
11 Kouprina, N. & Larionov, V. Innovation - TAR cloning: insights into gene function, long-range haplotypes and genome structure and evolution. *Nature Reviews Genetics* **7**, 805-812, doi:10.1038/nrg1943 (2006).
12 Kouprina, N., Noskov, V. N. & Larionov, V. Selective isolation of large chromosomal regions by transformation-associated recombination cloning for structural and functional analysis of mammalian genomes. *Methods Mol Biol* **349**, 85-101, doi:10.1385/1-59745-158-4:85 (2006).
13 Gibson, D. G. *et al.* Enzymatic assembly of DNA molecules up to several hundred kilobases. *Nat Methods* **6**, 343-345, doi:10.1038/nmeth.1318 (2009).

Supplementary Note 2

Assembly of CFTR by BASIS

In the first step of BASIS we mixed "recipient cells" – containing a BAC with the first section of the CFTR gene followed by a +2/-2 double selection cassette, and a tetracycline resistance conferring plasmid (+5, tet^R) encoding arabinose-inducible lambda red components and Cas9 protein – with "donor cells". The donor cells contained a donor BAC encoding the second section of the CFTR gene followed by a +3/-3 double selection cassette (+3, hyg^R (confers growth on hygromycin) and a non-transferable F' plasmid. The donor BAC contains a -2 marker on the backbone.

We selected (in liquid media containing hygromycin and tetracycline) for recipient cells that had received the donor BAC, via conjugative transfer, and we turned on (with arabinose) the expression of the Cas9 protein and the lambda red recombination components from the helper plasmid in the recipient. Universal spacers (expressed from the donor BAC) combined with Cas9 led to the excision of the second section of the CFTR gene, and the adjacent +3/-3 double selection cassette and universal homology region from the donor BAC. The sequences at the ends of the excised linear DNA are homologous to the sequences flanking the +2/-2 marker on the assembly BAC. Lambda red-mediated recombination led to insertion of the second section of the CFTR gene and the +3/-3 double selection cassette in place of the +2/-2 selection cassette in the assembly BAC.

We selected, on agar plates containing tetracycline, hygromycin, and sucrose, for recipient cells that had acquired the positive selection marker (+3, hyg^R) from the donor BAC, lost the backbone of the donor BAC (-2, *sacB*), and lost the negative selection marker (-2, *sacB*) from the assembly BAC. This resulted in selection for cells containing an intermediate BAC, which encodes section one and two of the CFTR gene followed by a +3/-3 selection cassette and a tetracycline resistance conferring plasmid (+5, tet^R), encoding arabinose-inducible lambda red components and Cas9. Only clones with the correct phenotype for each of the markers exchanged via BASIS (+2, -2, +3, -3) were sequenced (**Extended Data Fig. 3b, Methods**). Cells containing a correctly sequenced clone were used as "recipient cells" for the second step of BASIS.

We mixed the recipient cells for the second step of BASIS with "donor cells" containing a donor BAC, encoding the third section of the CFTR gene followed by a +2/-2 double selection cassette, and a non-transferable F' plasmid. The donor BAC contains a -1 marker on the backbone. We selected (in liquid media containing chloramphenicol and tetracycline) for recipient cells that have received the donor BAC via conjugative transfer and turned on (with arabinose) the expression of the Cas9 protein and the lambda red recombination components from the helper plasmid in the recipient. Universal spacers (expressed from the donor BAC) combined with Cas9 led to the excision of the third section of the CFTR gene and the adjacent +2/-2 double selection cassette and universal homology region from the donor BAC. The sequences at the ends of the excised linear DNA are homologous to the sequences flanking the +3/-3 marker on the intermediate BAC. Lambda red-mediated recombination leads to the insertion of the third section of the CFTR gene and the +2/-2 double selection cassette in place of the +3/-3 selection cassette in the assembly BAC.

We selected, on agar plates containing tetracycline, chloramphenicol, streptomycin, and 4-chlorophenylalanine, for recipient cells that had acquired the positive selection marker

(+2, *cat*) from the donor BAC, lost the backbone of the donor BAC (-1, *rpsL*), and lost the negative selection marker (-3, *pheS**) from the assembly BAC. This resulted in selection for cells containing a final BAC encoding the whole CFTR gene followed by a +2/-2 cassette.

Fig.1 c

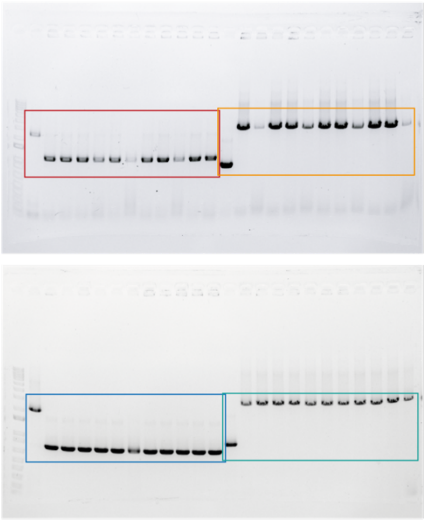

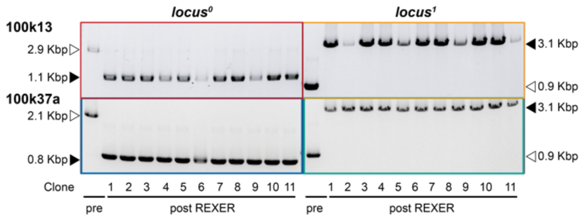

Extended Data Fig. 6 c

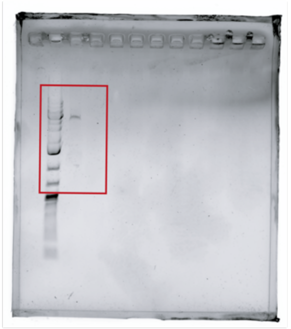

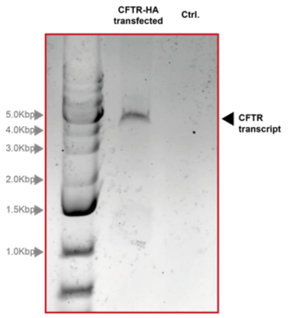

Supplementary Fig. 2 b

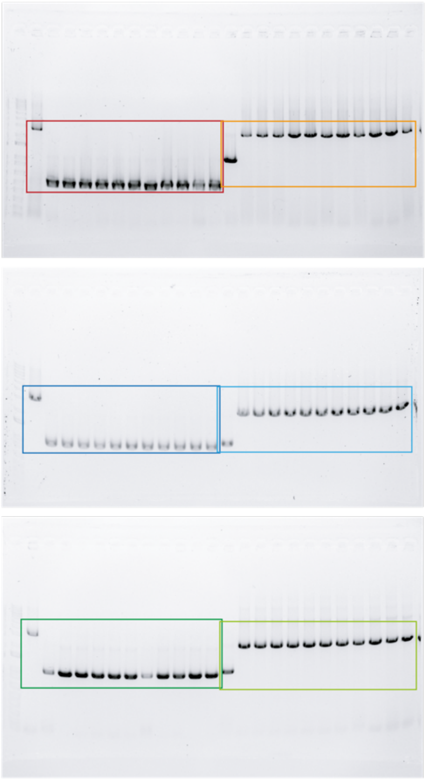

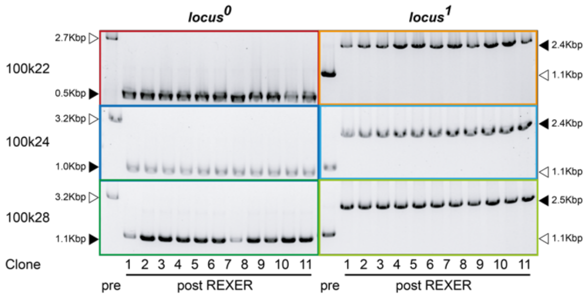

Supplementary Fig. 1 | Gel image source files

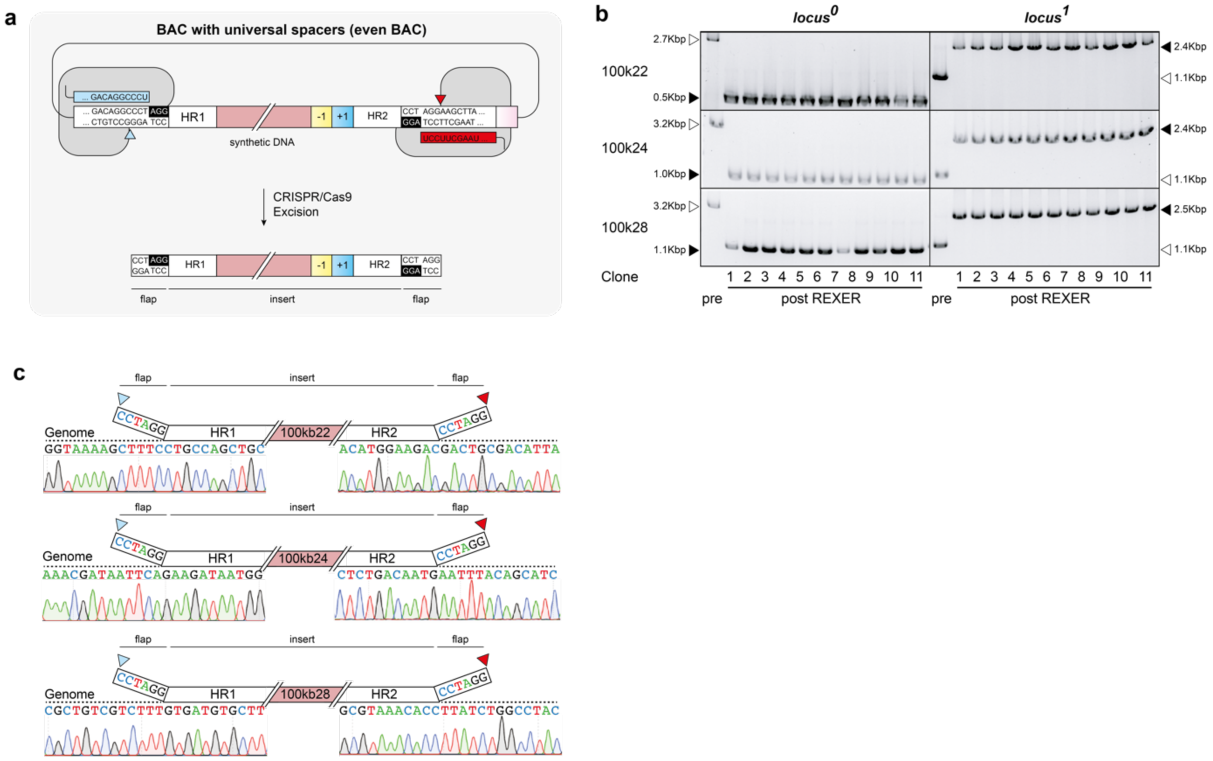

Supplementary Fig. 2 | REXER with universal spacers results in scarless genomic integration of large synthetic DNA fragments – even numbered examples.

a, Two BAC backbones (even and odd) were used for total synthesis of the *E. coli* genome. The BAC shown here is even numbered. The architecture for odd numbered BACs is shown in the main text (**Fig 1b**). Even and odd BACs contain distinct positive and negative selection cassettes, which allows REXER to be iterated. We designed Universal1 spacers for all odd numbered BACs and Universal2 spacers for all even numbered BACs (**Supplementary Data 1**). One universal spacer RNA (blue) targets the sequence in the BAC backbones 5’ to the insert; this sequence is common to both backbones. The BAC backbone sequences 3’ to the insert are distinct in the two BAC backbones, and therefore two different universal spacer sequences (yellow and red, respectively) were designed (**Fig 1b**). Cut sites are indicated with coloured triangles, PAM sequences are shown in black boxes, selection cassettes are shown in coloured arrows, and synthetic DNA is shown in pink. **b**, Verification by genotyping of the 5’ and 3’ genomic integration sites after REXER using universal spacers at five genomic loci. At the 5’ $locus^0$, a double selection cassette is removed from the genome upon successful replacement by REXER, while another double selection cassette is inserted at the 3’ $locus^1$. 11 post-REXER clones were genotyped for each experiment. Triangles indicate the size of the expected PCR product at each locus before (white) and after (black) REXER. **c**, Sequence verification of the ends of the integration sites after REXER using universal spacer RNA. The excised synthetic DNA flanked by HRs and 6 bp of non-homologous sequence (tilted) is shown above the sequence that is expected for scarless integration. Five post-REXER clones were sequenced for each experiment. We did not observe integration of the non-homologous termini for any clone and neither did any point mutations appear (Full data set in **Supplementary Fig. 3**). The experiments in **b** were performed in one biological replicate.

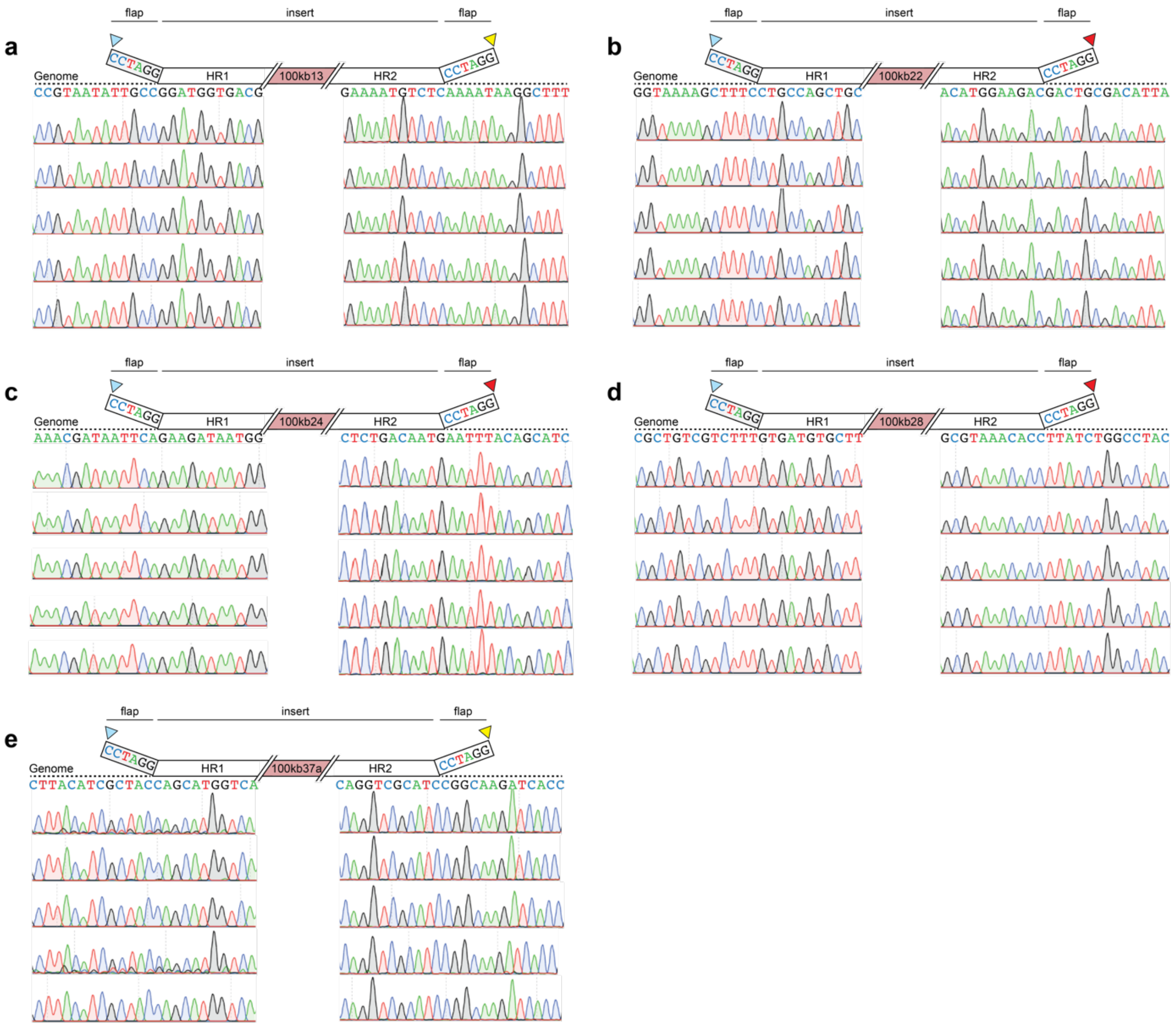

Supplementary Fig. 3 | Sanger sequencing of integration sites after REXER with universal spacers.

Two sets of universal spacer RNAs were used for REXER experiments targeting five loci across the genome **(Fig. 1d, Supplementary Fig. 2c)**. The genomic integration sites were verified by Sanger sequencing of five individual colonies of (a) 100k13, (b) 100k22, (c) 100k24, (d) 100k28, and (e) 100k37a. Despite the excised synthetic DNA being flanked by 6 bp of non-homologous sequences (tilted), in no instance did these termini interfere with scarless integration.

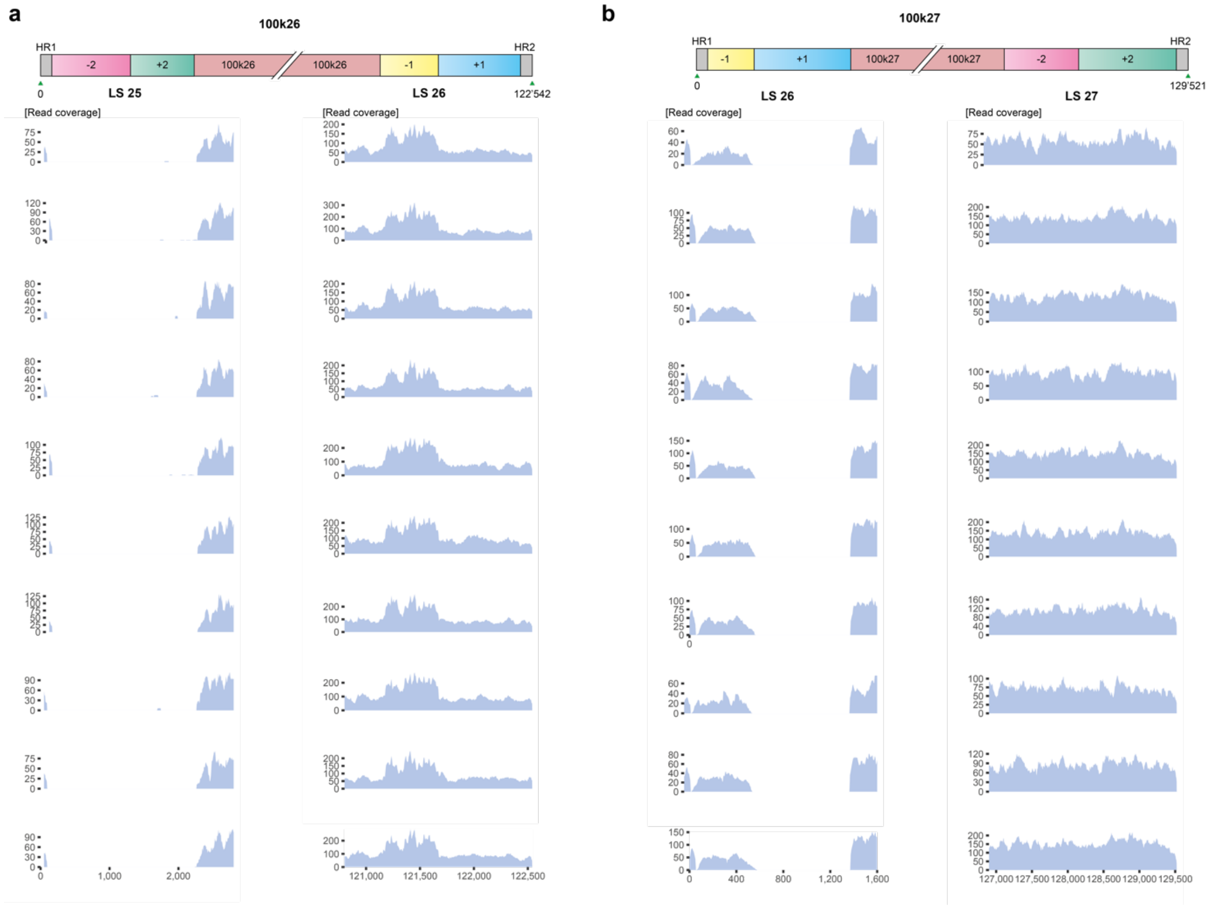

Supplementary Fig. 4 | Sequencing post-CONEXER clone marker swap.
a, Sequencing marker swap in post-CONEXER clones, following CONEXER with BAC 100k26. The y axis shows read coverage from Illumina sequencing. All clones sequenced had the correct set of post-CONEXER phenotypes (**Extended Data Fig. 2c**). Sequence analysis confirms loss of the +2/-2 marker (*sacB-cat*) at position 100-2’266 from the genome and integration of the +1/-1 marker (*rpsL-kan*^{R}) at position 121’161-122’479. Increased coverage at 121’299-121’673 stems from the endogenous copy of *rpsL* in the genome that has a K43R mutation. Positions are numbered from the beginning of HR1 to the end of HR2 (from 0 to 122’542). **b,** Sequencing marker swap in post-CONEXER clones, following CONEXER with BAC 100k27. The y axis shows read coverage from Illumina sequencing. All clones sequenced had the correct set of post-CONEXER phenotypes (**Extended Data Fig. 2c**). Sequence analysis confirms loss of the +1/-1 marker (*rpsL-kan*^{R}) at position 56-1’319 from the genome and integration of the +2/-2 (*sacB-cat*) marker at position 127’298-129’463. Coverage at 194-375 stems from the endogenous copy of rpsL in the genome that has a K43R mutation. Positions are numbered from the beginning of HR1 to the end of HR2 (from 0 to 129’521).

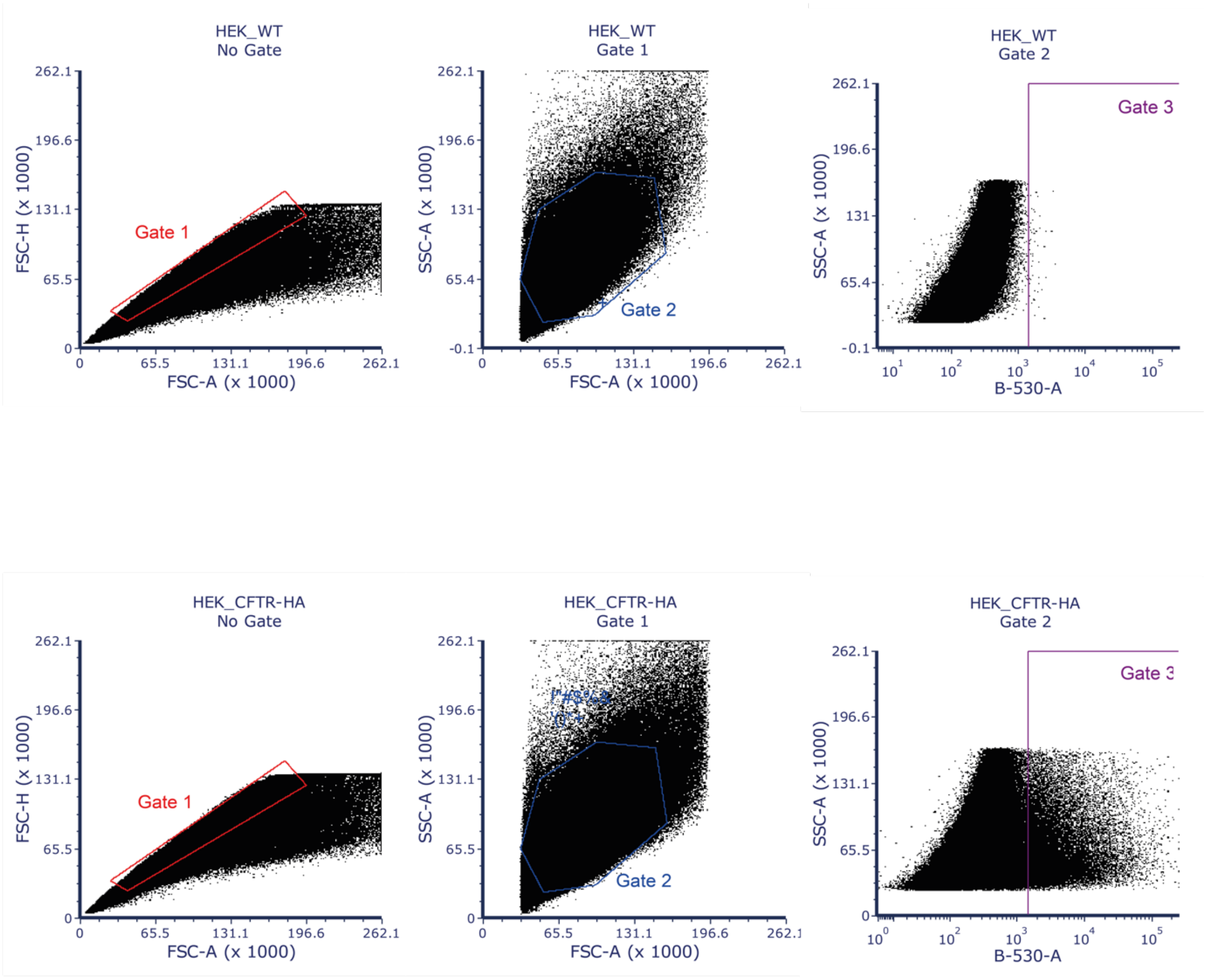

Supplementary Fig. 5 | FACS gating strategy for the CFTR BASIS BAC transfection. The GFP-positive cells were sorted on the third day post-transfection and used for RNA isolation and cDNA preparation. The gates were defined on FSC-A and FSC-H for single cells (Gate 1) and on FSC-A and SSC-A for the desired cell population (Gate 2). We defined the GFP gate (Gate 3) based on the negative control of untransfected HEK293 cells.

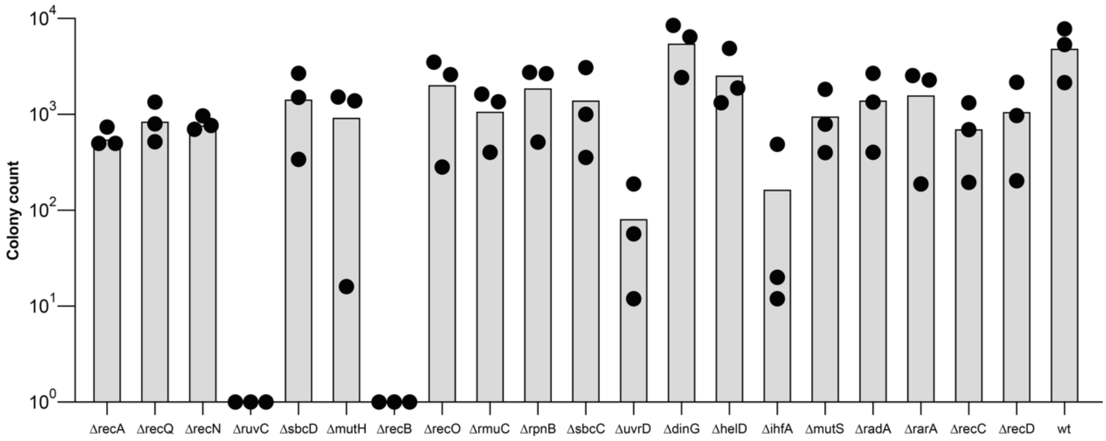

Supplementary Fig. 6 | Colony count for CONEXER mediated genomic replacement in different genomic backgrounds.

Screening of single gene deletion strains in CONEXER mediated replacement of genomic section 100k24. Colony counts obtained in each experiment are indicated. Deletion of recA leads to a reduction of colony count. Bars represent the mean of n=3 biological replicates.
